# Performance Analyses of a RAIM Algorithm for Kalman Filter with GPS and NavIC Constellations

**DOI:** 10.3390/s21248441

**Published:** 2021-12-17

**Authors:** Susmita Bhattacharyya

**Affiliations:** Department of Aerospace Engineering, Indian Institute of Technology Kharagpur, Kharagpur 721302, India; susmita@aero.iitkgp.ac.in

**Keywords:** Kalman filter, GNSS, RAIM, Schmidt Kalman filter, weighted least squares

## Abstract

This paper evaluates the performance of an integrity monitoring algorithm of global navigation satellite systems (GNSS) for the Kalman filter (KF), termed KF receiver autonomous integrity monitoring (RAIM). The algorithm checks measurement inconsistencies in the range domain and requires Schmidt KF (SKF) as the navigation processor. First, realistic carrier-smoothed pseudorange measurement error models of GNSS are integrated into KF RAIM, overcoming an important limitation of prior work. More precisely, the error covariance matrix for fault detection is modified to capture the temporal variations of individual errors with different time constants. Uncertainties of the model parameters are also taken into account. Performance of the modified KF RAIM is then analyzed with the simulated signals of the global positioning system and navigation with Indian constellation for different phases of aircraft flight. Weighted least squares (WLS) RAIM used for comparison purposes is shown to have lower protection levels. This work, however, is important because KF-based integrity monitors are required to ensure the reliability of advanced navigation methods, such as multi-sensor integration and vector receivers. A key finding of the performance analyses is as follows. Innovation-based tests with an extended KF navigation processor confuse slow ramp faults with residual measurement errors that the filter estimates, leading to missed detection. RAIM with SKF, on the other hand, can successfully detect such faults. Thus, it offers a promising solution to developing KF integrity monitoring algorithms in the range domain. The modified KF RAIM completes processing in time on a low-end computer. Some salient features are also studied to gain insights into its working principles.

## 1. Introduction

Since its inception, satellite navigation has become a critical infrastructure that supports a significant part of everyday life today. Its diverse array of applications includes power grid synchronization, precision farming, and emergency services, to name a few [1]. Although the system keeps the modern world running, it is vulnerable to numerous sources of signal degradation, anomalies, and intentional or unintentional interference. It is estimated that a failure of the global navigation satellite systems (GNSS)—a collective term for different satellite navigation systems—could cost modern economies billions of dollars [2]. Furthermore, growing reliance on GNSS for safety- and liability-critical applications, such as civil aviation and autonomous navigation, requires adherence to stringent performance standards for continuity and reliability of services. Faults or anomalies without timely detection can lead to hazardous consequences in such operations. Potential failure modes of GNSS for precise positioning are discussed in detail in [3].

While the performance of the global positioning system (GPS) has improved since 2012 with a lower number of fault counts [4], continuous monitoring is required by the constellation service provider as well as at the user level for high-reliability applications. Newer constellations (e.g., navigation with Indian constellation (NavIC) of India [5]) would possibly need several years to reach a state with few observed faults, thus requiring constant monitoring. Integrity is considered an important performance metric for operations that demand high reliability. It quantifies the level of trust placed in GNSS positioning. Receiver autonomous integrity monitoring (RAIM) is a GNSS receiver-based algorithm implemented in the user equipment to monitor integrity. It carries out consistency checks to detect faults and outputs position error bounds or protection levels (PL) when no anomaly is found. If an anomalous behavior is detected, the system needs to issue an alert to the user within a pre-determined time called the time to alert to avoid misleading information.

RAIM can be broadly classified into two categories depending on the standard implementations of the navigation processor of the receiver—weighted least squares (WLS) RAIM and Kalman filter (KF) RAIM. There exists a substantial amount of research on WLS RAIM [6,7,8]. In contrast, KF RAIM is much less explored [9]. KF is considered an integral component of advanced navigation algorithms. Examples are precise point positioning with GNSS [10], vector receiver architecture [11], and multi-sensor integration [12]. KF is a preferred choice in these systems for its ability to combine dynamics or a priori information with measurements and to estimate residual sensor errors [13]. The aforementioned advanced methods are shown to enhance performance by either improving accuracy, tracking high dynamics, or bridging GNSS outages in challenging environments. In order to ensure reliability along with these desirable features, integrity monitoring is an essential step. As a result, developing KF RAIM algorithms has received significant attention in recent years. A literature survey of contemporary work on KF-based integrity monitoring is provided next.

Reference [9] reviews different sensor integration architectures for intelligent transportation systems and state-of-the-art methods for fault detection and exclusion (FDE) and PL calculations. In this context, all KF-based methods up to the time of writing are also discussed. In [14], an FDE method is developed for the tight coupling of GNSS and inertial navigation system (INS) to detect multiple GNSS as well as GNSS and INS faults. It uses two detectors to separate GNSS and INS faults and a two-step fault exclusion process. A no subset advanced RAIM (ARAIM) algorithm is derived in [15] for the tight integration of GNSS and INS under some assumptions. It is shown to reduce the computational load to a great extent and provide global coverage for CAT I precision approaches. Reference [16] designs a computationally efficient KF RAIM algorithm for GNSS receivers. It checks measurement inconsistencies in the range domain, models time correlated errors, and requires Schmidt KF (SKF) as the navigation processor. A KF integrity monitoring algorithm is proposed in [17] for robot localization. It monitors faults within a time window with innovation-based test statistics and is also robust against previous faults. In [10] a navigation system of GNSS, INS, and an odometer is developed, and tight PLs are produced with ARAIM for precise point positioning. A parallel processing strategy coupled with the efficient software design of [18] shows that real-time operations of a substantially large number of KF banks are feasible on a GPU for integrity monitoring.

In [19], an FDE scheme is presented with the colored noise of KF represented as a first order autoregressive model. Fault detection tests for a real-time kinematic application show improved FDE performance and lower false alarm rates. An adaptive interactive multiple model framework is described in [12] to switch between Huber KF and RAIM for robustness and reliability. The proposed model offers better positioning performance than individual sub-models for a relative navigation system with GNSS and ultra wideband observations. Reference [20] discusses a Kalman integrated PL approach with empirically tuned parameters. It demonstrates better performance of the new approach over two other methods for an autonomous vehicle state estimation with GNSS, INS, and an odometer. A sliding window-based FDE method is proposed in [21] for integrated GNSS–INS. It is shown to improve positioning accuracy over that without fault exclusion. RAIM algorithms for advanced nonlinear filters [22] and vector receivers [23] are also described in the literature.

Five of the aforementioned references discuss KF-based PL calculations. Among them, [10,15] perform fault detection in the position domain, [16,17] follow range domain methods, and [20] assumes that fault detection and mitigation have been executed. This paper attempts to explore range domain methods further for their relatively low architectural complexity. To this end, it extends the work in [16], which uses an SKF [24] navigation processor and carrier-smoothed pseudoranges for absolute positioning. SKF accounts for measurement errors as ”consider parameters” without estimating them. As explained in the prior work, it allows the formation of two simultaneous fault detection tests, each with terms of a finite number of epochs. This ensures that the KF PLs do not grow with time. However, the methodology assumes a 100-second time constant for all errors, which is unrealistic, and hence, an important limitation of the work. In addition, the algorithm is validated with simulated range and range rate measurements, not generated by processing GNSS signals with a receiver. Therefore, the measurements do not properly represent the carrier smoothing of code. Fault detection performance is also not analyzed.

In order to address the above shortcomings, first, realistic error models of the carrier-smoothed pseudorange measurements are integrated into KF RAIM. More precisely, the error covariance matrix for fault detection is modified to capture the temporal variations of individual errors with different time constants. Uncertainties of the model parameters are also taken into account. Performance of the modified KF RAIM is then evaluated with the simulated signals of GPS and NavIC for aircraft navigation during different phases of flight. Intermediate frequency (IF) data are post-processed by a developed GPS L1-NavIC L5 software receiver to generate carrier-smoothed code and precise range rate measurements for RAIM. Some salient features of KF RAIM are also studied to gain insights into its working principles.

Performance analyses show that WLS RAIM [25] used for comparison purposes has lower PLs than the KF RAIM. However, WLS is not suitable for advanced navigation methods, such as multi-sensor integration and vector receivers, where KF is an integral part. Therefore, KF-based integrity monitors are essential. An important finding of the analyses is as follows. Innovation-based tests with an extended KF (EKF) navigation processor mistake slow ramp for the residual measurement error that the filter estimates, leading to missed detection. RAIM with SKF, on the other hand, can successfully detect such faults. It also completes processing in time on a low-end computer. Thus, it offers a promising solution to developing KF RAIM in the range domain. The current work does not consider constellation-wide faults. Measurements from different satellites at a particular time epoch are assumed to be uncorrelated with one another.

The remaining paper is organized as follows. First, the KF RAIM extended in this paper is briefly outlined for ease of understanding of the subsequent modifications. Next, extensions of the algorithm are presented in detail. Following this, simulation studies are performed. Finally, the paper concludes with a summary of the key findings and future work.

## 2. Overview of Existing KF RAIM

Both fault detection test statistics and PLs of KF RAIM presented in [16] are briefly discussed in this section for ease of understanding of the subsequent developments.

### 2.1. Fault Detection Test Statistics

Assuming only white noise in the GNSS measurements, three fault detection test statistics ($\alpha_{j,\;k}$; *j* = 1,…,3) at $t_{k}$ are formulated in [26] as

(1) $$\alpha_{j,\;k}^{2}=\sum_{\ell =p_{j,\;k}}^{m_{j,\;k}}\left(\Delta {\underline{\rho }}_{\ell }^{T}W_{\ell }^{-1}\left(I-G_{\ell }\right)\Delta {\underline{\rho }}_{\ell }+\Delta {\underline{\dot{\rho }}}_{\ell }^{T}{W_{rr,\;\ell }}^{-1}\left(I-G_{\ell }^{rr}\right)\Delta {\underline{\dot{\rho }}}_{\ell }\right)$$

where I represents the identity matrix of dimension n×n. *n* is the number of visible satellites at a given epoch and can change with time. $\Delta {\underline{\rho }}_{\ell }$ and $\Delta {\underline{\dot{\rho }}}_{\ell }$ are pseudorange and pseudorange rate innovation vectors of the EKF, respectively, at time epoch *ℓ*. The limits of *ℓ* on the right hand side, $m_{j,\;k}$ and $p_{j,\;k}$, change with *j* and *k*. $G_{\ell }$ and $G_{\ell }^{rr}$ are given by WLS estimation as

(2) $$G_{\ell }=H_{\ell }{\left(H_{\ell }^{T}W_{\ell }^{-1}H_{\ell }\right)}^{-1}H_{\ell }^{T}W_{\ell }^{-1}$$

(3) $$G_{\ell }^{rr}=H_{\ell }{\left(H_{\ell }^{T}{W_{rr,\;\ell }}^{-1}H_{\ell }\right)}^{-1}H_{\ell }^{T}{W_{rr,\;\ell }}^{-1}$$

where $H_{\ell }$ is the linearized measurement model matrix for pseudoranges (or pseudorange rates) and of dimension n×4 for a single constellation and n×5 for dual constellations. $W_{\ell }$ is the error covariance matrix of the pseudorange measurements at $t_{\ell }$. $W_{rr,\;\ell }$ is its counterpart for pseudorange rates.

WLS estimation-based fault detection in KF is used because it helps in the following ways. It results in the noise statistics of αs being easily computed. Furthermore, each term under the summation of Equation (1) contributes the fault of its own epoch only, not of any other epochs. Thus, the computation of the failure mode slope (FMS) for mean position error bound is simplified. In addition, each α can have a separate FMS. Consequently, three tests can be formulated. With three suitably designed test statistics, the PL is shown not to increase with time in [26], as opposed to the PL with a single statistic.

The limitation of the above formulation is that measurement errors across time epochs are assumed to be uncorrelated, which is a gross simplification. To overcome this limitation, [16] assumes the following error models. Broadcast satellite orbit and clock error, residual tropospheric error, receiver noise, and multipath are together represented as a first order Gauss Markov (FOGM) random process with a 100 s time constant (carrier smoothing interval of code). From [27], individual error standard deviations are obtained for GPS carrier smoothed code for airborne users. The second constellation considered is NavIC. As for NavIC, the same equations are used with frequency bands properly changed. This is because equivalent error models are yet to be developed for this constellation. No ionospheric error is modeled, which can be eliminated with dual frequency measurements. Only white noise pertaining to 45 dB-Hz C/N${}_{0}$ is assumed to be present in the pseudorange rate measurements.

Considering time-correlated errors in the carrier-smoothed pseudorange measurements, and dropping time instant *k* for simplicity of notation, the first part of Equation (1) for an epoch changes to

(4) $$\begin{array}{c} {\left(\alpha_{j}^{\prime }\right)}^{2}={\Delta {\underline{\tilde{\rho }}}^{*}}^{T}{\tilde{S}}^{\prime }{\tilde{S}}^{\prime T}\Theta {\tilde{S}}^{\prime }{\tilde{S}}^{\prime T}\Delta {\underline{\tilde{\rho }}}^{*} \end{array}$$

where $\Delta {\underline{\tilde{\rho }}}^{*}$ stacks all $\Delta {\underline{\rho }}_{\ell }^{*}$ vectors given by $\Delta {\underline{\rho }}_{\ell }^{*}=W_{\ell }^{-1/2}\Delta {\underline{\rho }}_{\ell }$, *ℓ* varying from $p_{j}$ to $m_{j}$; $\Delta {\underline{\rho }}_{\ell }$ denotes the carrier-smoothed pseudorange innovation vector at epoch *ℓ*; and $W_{\ell }$ is its error covariance. ${\tilde{S}}^{\prime }$ is a full rank block matrix, with its *ℓ*th block along the diagonal given by $S_{\ell }^{\prime }$, where $\left(I-G_{\ell }^{*}\right)=S_{\ell }^{\prime }{S_{\ell }^{\prime }}^{T}$ and $G_{\ell }^{*}=W_{\ell }^{-1/2}H_{\ell }{\left(H_{\ell }^{T}W_{\ell }^{-1}H_{\ell }\right)}^{-1}\times H_{\ell }^{T}W_{\ell }^{-1/2}$, and other elements are zero. Θ is a square matrix with dimension $\left(\sum_{\ell =p_{j}}^{m_{j}}n\right)\times \left(\sum_{\ell =p_{j}}^{m_{j}}n\right)$. ’×’ represents the multiplication operation. ${\tilde{S}}^{\prime }{\tilde{S}}^{\prime T}$ on either side of Θ in Equation (4) ensures that only the *ℓ*th epoch’s fault is included from ${\Delta {\underline{\rho }}^{*}}_{\ell }$.

In order to perform fault detection tests with ${\left(\alpha_{j}^{\prime }\right)}^{2}$, SKF implementation [28] is used in the navigation processor. SKF offers reasonably good performance, while being computationally efficient. It models time-correlated errors without estimating additional states. As a result, these errors are not changed in the pseudorange innovations. Thus, SKF along with the help of ${\tilde{S}}^{\prime }{\tilde{S}}^{\prime T}$ allows a separate FMS for each of the test statistics. Hence, multiple test statistics can be formed. Each is with a given number of epochs.

Equation (4) is finally reduced to the form given by

(5) $${\left(\alpha_{j}^{\prime }\right)}^{2}={{\underline{\eta }}_{r}}^{T}\Theta_{2}{\underline{\eta }}_{r}$$

where ${\underline{\eta }}_{r}$ consists of unit-variance independent elements. Matrix $\Theta_{2}$ is determined by ${\tilde{S}}^{\prime }$, Ψ (measurement error covariance matrix of $\Delta {\underline{\tilde{\rho }}}^{*}$), and Θ. Since the maximum eigenvalue of $\Theta_{2}$, $\lambda_{\Theta ,\;2}^{\max}$ is designed to be less than or equal to one, fault detection thresholds of ${\left(\alpha_{j}^{\prime }\right)}^{2}$ can be derived from the chi-squared distribution [29]. Θ is formulated such that $\lambda_{\Theta ,\;2}^{\max}$ is less than or equal to one. It should be noted that $\lambda_{\Theta ,\;2}^{\max}$ is also equal to the maximum eigenvalue of ${\tilde{S}}^{\prime T}\Psi {\tilde{S}}^{\prime }{\tilde{S}}^{\prime T}\Theta {\tilde{S}}^{\prime }$ (=Ω), $\lambda_{\Omega }^{\max}$.

Thus, the test statistics shown in Equation (1) are changed with time-correlated errors in the carrier-smoothed pseudorange measurements as

(6) $$\alpha_{j}^{2}={\Delta {\underline{\tilde{\rho }}}^{*}}^{T}{\tilde{S}}^{\prime }{\tilde{S}}^{\prime T}\Theta {\tilde{S}}^{\prime }{\tilde{S}}^{\prime T}\Delta {\underline{\tilde{\rho }}}^{*}+\sum_{\ell =p_{j}}^{m_{j}}\left(\Delta {\underline{\dot{\rho }}}_{\ell }^{T}{W_{rr,\;\ell }}^{-1}\left(I-G_{\ell }^{rr}\right)\Delta {\underline{\dot{\rho }}}_{\ell }\right)$$

where *j* = 1, 2. At an epoch, if $\alpha_{1}$ crosses its threshold $T_{th,\;1}$, or $\alpha_{2}$ is greater than its threshold $T_{th,\;2}$, or both occur, a fault is detected. The third test is not formulated. Necessary modifications for not performing the third test are made to PLs. $\alpha_{1}$ is formed with terms of *M* recent epochs including the current one, whereas $\alpha_{2}$ has terms of (N−M) previous epochs. Both *M* and *N* are calculated adaptively at each time epoch. The matrices Ψ and Θ are briefly described next.

Ψ can be re-arranged into a block diagonal matrix, with *i*th block for satellite *i* given as $\Psi_{\mathrm{sub}}^{i}=\left[\begin{array}{ccccc} 1 & a_{11} & a_{11}a_{21} & a_{11}a_{21}a_{31} & \cdots \\ a_{11} & 1 & a_{21} & a_{21}a_{31} & \cdots \\ a_{11}a_{21} & a_{21} & 1 & a_{31} & \cdots \\ & & \vdots & \end{array}\right]$, where $a_{\ell 1}$ at an epoch $t_{\ell }$ takes a value between $a_{\min}$ and $a_{\max}$; $a_{\min}$ = 0.9890; and $a_{\max}$ = 0.9911 with a 5° elevation mask angle. In this context, another matrix $\Psi_{\max}$ is also defined. It has the same structure as that of Ψ, but its sub-matrix $\Psi_{\mathrm{sub},\;\max}^{i}$ is formed with $a_{\ell 1}$ always set as $a_{\max}$. Θ is given by ${\left(cI+\Psi \right)}^{-1}$ for $\alpha_{1}^{\prime }$, with *c* being a positive multiplication factor calculated adaptively to ensure that $\lambda_{\Theta ,\;2}^{\max}$ is less than or equal to one. For $\alpha_{2}^{\prime }$, Θ is ${\left(cI+\Psi_{\max}\right)}^{-1}$, with *c* as $\left(\lambda_{\Psi ,\;\max}^{\max}-\lambda_{\Psi ,\;\max}^{\min}\right)$. $\lambda_{\Psi ,\;\max}^{\max}$ and $\lambda_{\Psi ,\;\max}^{\min}$ are the maximum and minimum eigenvalues of $\Psi_{\max}$.

### 2.2. Protection Levels (PLs)

The SKF estimates error in the a priori user position and velocity in the Earth Centered Earth Fixed (ECEF) frame, and the receiver clock offsets and drifts for GPS and NavIC from respective truths. Furthermore, the time correlated error of each carrier-smoothed pseudorange measurement is modeled as an FOGM process but not estimated by the SKF. First, mean position error bounds are discussed, and then PL equations are provided.

#### 2.2.1. Mean Position Error Bounds

The mean position error, ${\underline{\mu }}_{k}^{\prime }$, of the navigation filter at epoch $t_{k}$ in the local navigation frame can be written as

(7) $${\underline{\mu }}_{k}^{\prime }=-\underset{\mathrm{Ignored}\;\mathrm{terms}}{\underbrace{\sum_{j=N-1}^{k-(m+1)}B_{k-j}^{\prime }{\underline{f}}_{k-m-j}}}\;-\underset{N\mathrm{terms}\;\mathrm{corresponding}\;\mathrm{to}\;\alpha_{1}\;\&\;\alpha_{2}}{\underbrace{\left(\sum_{j=0}^{N-2}B_{k-j}^{\prime }{\underline{f}}_{k-m-j}+K_{s,\;k}^{\prime }{\underline{f}}_{\;k-m+1}\right)}}$$

where $K_{s,\;k}^{\prime }$ = $C_{e}^{n}K_{s,\;k}\left(1:2:5,:\right)$ and $B_{k-j}^{\prime }$ = $C_{e}^{n}B_{k-j}\left(1:2:5,:\right)$. $B_{k-j}$ = $\left[\prod_{\ell =k}^{k-j}A_{\ell }\right]K_{s,\;k-j-1}$, and $A_{\ell }\;=\;\left(I-K_{s,\;\ell }C_{s,\;\ell }\right)F_{s,\;\ell -1}$. $F_{s}$, $C_{s}$, and $K_{s}$ are the state transition, measurement model, and Kalman gain matrices of SKF for the estimated states. For a matrix J, J(1:2:5,:) comprises the first, third, and fifth rows of J. In $B_{k-j}$ and $K_{s,\;k}$, these rows belong to the position states of the filter. $C_{e}^{n}$ is the coordinate transformation matrix from ECEF to the north-east-down (NED) frame. The two test statistics, $\alpha_{1}$ and $\alpha_{2}$, together have terms of *N* recent epochs. Terms from other previous epochs are ignored. An upper bound of the contributions from these terms to the mean position error is derived mathematically. Fault is represented by a vector ${\underline{f}}_{k-m+1}$ at $t_{k}$; it started at epoch *m*. Three or more simultaneous faults are not required to be monitored for the integrity risk considered. If $s_{1}$ and $s_{2}$ represent any two faulty satellite indices, then ${\underline{f}}_{k-m+1}\left(s_{1}\right)=b_{1,\;k-m+1}$, ${\underline{f}}_{k-m+1}\left(s_{1}+n\right)={\dot{b}}_{1,\;k-m+1}$, ${\underline{f}}_{k-m+1}\left(s_{2}\right)=b_{2,\;k-m+1}$, and ${\underline{f}}_{k-m+1}\left(s_{2}+n\right)={\dot{b}}_{2,\;k-m+1}$, with all other elements being zero. For single fault (SF) cases, only the $s_{1}th$ satellite has a fault. The mean error bound of the vertical position for $\alpha_{1}$ under a fault mode *i* (which can be SF or dual faults (DF), with *i* = $1,\ldots ,N_{fault}$), $VPE_{1,\;i}^{U}$ is obtained as follows.

For a fault mode, the square of the maximum FMS [25] for $\alpha_{1}$ (having *M* recent epoch terms from $t_{k}$ to $t_{k-M+1}$) is

(8) $$(\mathrm{maximum}\;\;{\mathrm{FMS})}_{\alpha 1,\;i}^{2}=\max_{{\underline{f}}_{w}^{T}\Lambda {\underline{f}}_{w}\;=\;\Gamma }\frac{{\underline{f}}_{w}^{T}L{\underline{f}}_{w}}{{\underline{f}}_{w}^{T}\Lambda {\underline{f}}_{w}}$$

The FMS is maximized with the worst-case fault vector, ${\underline{f}}_{w}$. L is formed with the appropriate elements of $B^{\prime }$ matrices over *M* recent epochs for a fault mode. ${\underline{f}}_{w}$ constitutes the corresponding *b* and $\dot{b}$ terms. Λ is

(9) $$\begin{array}{cc} \Lambda = & \left[\begin{array}{cc} \Theta_{3} & \left[0\right]\\ \left[0\right] & \Theta_{4} \end{array}\right] \end{array}$$

where [0] denotes a matrix of all zero entries. $\Theta_{3}$ is determined with the terms of Equation (4) corresponding to a fault mode. $\Theta_{4}$ is obtained from the second term of Equation (6) on the right. It is a diagonal matrix for SF modes and a block diagonal matrix with each block of size 2 × 2 for DF modes. A computationally efficient method is used to calculate the inverse of each block of $\Theta_{4}$ without inverting it. If $\lambda_{L\Lambda^{-1}}^{\max}$ is the maximum eigenvalue of $L\Lambda^{-1}$,

(10) $${\mathrm{VPE}}_{1,\;i}^{U}=\sqrt{\lambda_{L\Lambda^{-1}}^{\max}\Gamma }$$

where Γ is calculated as follows:

(11) $$\begin{array}{cc} {\left(T_{th,\;1}^{*}\right)}^{2} & =\frac{\lambda_{\tilde{\Theta }}^{\max}}{{\left(\lambda_{\tilde{\Theta }}^{\min}\right)}^{2}}T_{th,\;1}^{2};\sqrt{\Gamma^{*}}=\frac{1}{\sqrt{\lambda_{\tilde{\Theta }}^{\max}}}\left(\sqrt{\Gamma }-T_{th,\;1}\right)+T_{th,\;1}^{*} \end{array}$$

where $\lambda_{\tilde{\Theta }}^{\min}$ and $\lambda_{\tilde{\Theta }}^{\max}$ are the minimum and maximum eigenvalues of $\tilde{\Theta }$. The block diagonal matrix, $\tilde{\Theta }$, has two blocks. The first one is $\Theta_{2}$ (see Equation (5)), and the second one is the identity matrix. Hence, $\lambda_{\tilde{\Theta }}^{\max}$ = 1, and $\lambda_{\tilde{\Theta }}^{\min}$ = $\lambda_{\Theta ,\;2}^{\min}$ (minimum eigenvalue of $\Theta_{2}$), which is shown to be equal to the minimum eigenvalue of Ω, $\lambda_{\Omega }^{\min}$. It is also proved that all eigenvalues of Ω are real and positive. First, $\Gamma^{*}$ is determined such that the probability of the non-central chi-squared distribution $P_{ncx}\left({\left(T_{th,\;1}^{*}\right)}^{2},\mathrm{DOF},\Gamma^{*}\right)$ = $P_{MD,\;i}^{alloc}$; DOF stands for the required degrees of freedom. $P_{MD,\;i}^{alloc}$ is the allocated probability of missed detection for the *i*th fault mode. Then, Γ is calculated from Equation (11) with $\lambda_{\tilde{\Theta }}^{\max}$ = 1 and saved in a lookup table for different values of DOF, $P_{MD,\;i}^{alloc}$, and $1/\lambda_{\tilde{\Theta }}^{\min}$. Following [30], it is also justified that Γ increases as $\lambda_{\tilde{\Theta }}^{\min}$ decreases. Γ for $\alpha_{1}$ is obtained from the lookup table for a fault mode, the next entry of $1/\lambda_{\tilde{\Theta }}^{\min}$ higher than the calculated value, and the required DOF.

$VPE_{2,\;i}^{U}$ for $\alpha_{2}$ is calculated similarly to that of $VPE_{1,\;i}^{U}$ but using a more computationally efficient approach. This is because it involves terms of (N−M) epochs, often found to be greater than *M*. ${VPE}_{3}^{U}$ is the mean vertical position error (VPE) upper bound for the ignored terms and the same for all fault modes. It is

(12) $${VPE}_{3}^{U}=U_{\max}N_{\max}\times \frac{1\times 10^{-6}}{1-0.1}\left({\hat{b}}_{\max}+{\dot{\hat{b}}}_{\max}\right)$$

In order to have the north and east components, the factor $1\times 10^{-6}$ is changed to $\sqrt{2}\times 10^{-6}$ for the horizontal position. The maximum of all *N*s up to the current epoch $t_{k}$ is denoted as $N_{\max}$. $U_{\max}$ is an upper bound obtained in a computationally efficient way at each epoch [26]. The maximum bias ${\hat{b}}_{\max}$ at epoch *k* is given as

(13) $$\begin{array}{cc} \left(\min_{\forall \;\ell =1,\ldots ,k}\min_{\alpha_{1},\;\alpha_{2}}\min_{i\in \mathrm{fault}\;\mathrm{modes}}\lambda_{\Theta ,\;3}^{\min}\right){\hat{b}}_{\max}^{2} & =\Gamma_{\max} \end{array}$$

where $\lambda_{\Theta ,\;3}^{\min}$ is the minimum eigenvalue of $\Theta_{3}$. Operation “min” represents the minimum of all terms underneath it. $\Gamma_{\max}$ is the maximum of all calculated Γs. The maximum bias rate, ${\dot{\hat{b}}}_{\max}$, can be determined in a similar way with the minimum eigenvalue of $\Theta_{4}$. $\Theta_{4}$ is a block-diagonal matrix, with a block having a dimension of one or two. It can be shown that for each two-dimensional block, the minimum eigenvalue is more than the ratio of its determinant to the sum of the diagonal elements. This lower bound of the minimum eigenvalue is used to find ${\dot{\hat{b}}}_{\max}$. Next, the PL equations are briefly mentioned.

#### 2.2.2. PLs

Equations for the vertical PL (VPL) are given as follows. They can be easily modified for the horizontal PL (HPL). Under the SF modes, VPL is

(14) $${\mathrm{VPL}}_{SF}=\sum_{j=1}^{2}\left(\max_{i\in \mathrm{SF}\;\mathrm{modes}}VPE_{j,\;i}^{U}\right)+VPE_{3}^{U}+\gamma_{s}\sigma_{v}$$

The operation “max” represents the maximum of all terms underneath it. The probability $P(\mathrm{absolute}\;\mathrm{VPE}>\gamma_{s}\sigma_{v})$ = $P_{MD,\;sf}^{alloc}$. $\sigma_{v}$ is the standard deviation of the error in the estimated vertical position. $P_{MD,\;sf}^{alloc}$ is the allocated probability of missed detection for SF modes. Similarly, the VPL under DF modes is calculated. The final VPL is

(15) $$\mathrm{VPL}=\max({\mathrm{VPL}}_{NF},\;{\mathrm{VPL}}_{SF},\;{\mathrm{VPL}}_{DF})$$

In the preceding equation, VPL${}_{NF}$ stands for the VPL under no fault conditions.

### 2.3. Limitations of Existing Approach and Modifications

The limitations of the described KF RAIM algorithm are as follows:

(1) SKF does not estimate user acceleration needed for moderate to high dynamics.
(2) Time constants of all time-correlated errors are assumed to be 100 s. More appropriate error models having different time constants for different error sources must be adopted.
(3) $a_{\ell 1}$ entries of $\Psi_{\mathrm{sub}}^{i}$ are calculated with modeled values of measurement error standard deviations. By definition, they should be obtained with true error standard deviations, which are not known. A more accurate model of $\Psi_{\mathrm{sub}}^{i}$ would therefore account for uncertainties in the knowledge of actual $a_{\ell 1}$.
(4) In the beginning of the receiver operation, relatively large transient position errors of the navigation filter can be handled by including artificial white noise in the carrier-smoothed pseudorange measurement error model of the SKF. While this can be easily incorporated in the SKF, the Ψ matrix of $\Delta {\underline{\tilde{\rho }}}^{*}$ does not model white noise.

Thus, Ψ needs to be appropriately modeled, considering more realistic error models, initial artificial white noise, and uncertainties in the knowledge of $a_{\ell 1}$. The next two sections discuss the adopted error models in the current work and appropriate bounds of the Ψ matrix. SKF formulation also estimates error in the a priori user acceleration from truth. *N* is adaptively calculated the same way as before with B matrices. *M* is also calculated as before, but with $B^{\prime \prime }$ matrices, where $B_{k}^{\prime \prime }$(1:2:5,:) = $B_{k}^{\prime }$ = $C_{e}^{n}B_{k}$(1:3:7,:), and $B_{k}^{\prime \prime }$(2:2:6,:) = $C_{e}^{n}B_{k}$(2:3:8,:). $B_{k}$(1:3:7,:) has rows of $B_{k}$ corresponding to the position states of the filter, and $B_{k}$(2:3:8,:) has rows for velocity states. This is found to reduce *M* and hence PLs. The effect of overbounding the pseudorange rate (or precise range rate) error covariance is also included, and a tighter value of Γ in Equation (11) is determined.

## 3. Modified Pseudorange Error Models and Ψ

In this section, first, the adopted error models of the carrier-smoothed pseudorange measurements with different time constants are explained. Following this, it is briefly discussed how they are incorporated in the SKF navigation filter. Finally, necessary modifications of $\Psi_{\mathrm{sub}}^{i}$ for satellite *i* are presented. It is assumed that the Ionospheric error is eliminated with dual frequency measurements. The following models are adopted for other error sources with dual frequency correction terms, when needed.

Residual zenith tropospheric delay after applying corrections of the MOPS model [31] has a latitude- and longitude-dependent bias, as noted in [32]. It can be corrected in the pseudorange measurements using a lookup table as a function of latitude and longitude. The remaining zenith error, ${\underline{\eta }}_{\mathrm{tr}}\left(i\right)$, is a zero-mean FOGM process with a standard deviation ($\sigma_{\mathrm{tr}}$) of 0.2 m and time constant ($\tau_{\mathrm{tr}}$) of 25 h for any visible satellite *i*. The broadcast ephemeris and satellite clock error, which is overbounded by the user range accuracy (URA), is denoted by ${\underline{\eta }}_{\mathrm{ec}}\left(i\right)$. It is modeled as an FOGM process, having a zero mean, unit standard deviation ($\sigma_{\mathrm{ura}}$), and 20 h time constant ($\tau_{\mathrm{ura}}$). The time between two effectively independent range error samples for satellite clock and orbit error is considered 50 h [33], which is taken as approximately 2.5 × the time constant. It is justified in [34] that common errors of code and carrier measurements remain unchanged in the carrier-smoothed code, thus allowing the same models to be used. As multipath and receiver noise are application specific, they are modeled for safety critical aviation applications in this paper. Their standard deviations, denoted as $\sigma_{\mathrm{ml},\;i}$ and $\sigma_{n,\;i}$, respectively, are obtained from [27] for airborne users. In addition, the constant term of the receiver noise standard deviation is inflated further to bound the autocorrelation function of carrier-smoothed pseudoranges due to multipath and noise derived in [35] for airborne users. The inflated value is 0.23 m in place of 0.15 m. Finally, multipath and receiver noise together (${\underline{\eta }}_{\mathrm{ml}-n}\left(i\right)$) are represented as a zero-mean FOGM process with a time constant ($\tau_{\mathrm{ml}-n}$) of 100 s and standard deviation $\sqrt{\sigma_{\mathrm{ml},\;i}^{2}+\sigma_{n,\;i}^{2}}$. In the current work, NavIC is considered the second constellation. The same models as those of GPS are adopted for it, but with frequency bands appropriately changed. The approach described in the paper also holds for other constellations with model parameters suitably changed.

The SKF navigation filter estimates error in the a priori position, velocity and acceleration of the user in ECEF coordinates, and receiver clock offsets and drifts for GPS and NavIC from respective truths. The states of the aforementioned FOGM processes for each visible satellite are the “consider parameters” of the SKF. The zenith tropospheric error is projected onto the line of sight (LOS) using the mapping function of the MOPS model in the measurement model matrix. In the simulation studies, the position error of the SKF is compared with that of an EKF that estimates all FOGM states to depict comparable performance. Since the SKF takes into account the effects of the “consider parameters” in its estimates, no additional terms other than those in Equation (14) are needed for PL calculation.

With modified error models, the $a_{\min}$ and $a_{\max}$ mentioned after Equation (6) for test statistics are re-calculated as follows. If $\underline{\eta }\left(i\right)$ is the carrier-smoothed pseudorange measurement error of satellite *i*, then it constitutes the following at an epoch *ℓ*:

(16) $${\underline{\eta }}^{\left(\ell \right)}\left(i\right)={\mathrm{mp}}^{\left(\ell \right)}\left(\theta^{\left(\ell \right)}\left(i\right)\right){\underline{\eta }}_{\mathrm{tr}}\left(i\right)+{\underline{\eta }}_{\mathrm{ec}}\left(i\right)+{\underline{\eta }}_{\mathrm{ml}-n}^{\left(\ell \right)}\left(i\right)$$

where the superscript with parentheses denotes time epochs, and mp(θ) is the mapping function of the MOPS model for elevation angle θ. The autocorrelation between ${\underline{\eta }}^{\left(\ell \right)}\left(i\right)$ and ${\underline{\eta }}^{\left(\ell +q\right)}\left(i\right)$ is

(17) $$\begin{array}{cc} E\left({\underline{\eta }}^{\left(\ell \right)}\left(i\right){\underline{\eta }}^{\left(\ell +q\right)}\left(i\right)\right) & ={\left(\sigma^{\left(\ell \right)}\left(i\right)\right)}^{2}\exp^{\left(-\beta q\Delta t\right)}={\mathrm{mp}}^{\left(\ell \right)}\left(\theta^{\left(\ell \right)}\left(i\right)\right){\mathrm{mp}}^{\left(\ell +q\right)}\left(\theta^{\left(\ell +q\right)}\left(i\right)\right)\sigma_{\mathrm{tr}}^{2}\exp^{\left(-q\Delta t/\tau_{\mathrm{tr}}\right)}+\\ \; & \sigma_{\mathrm{ura}}^{2}\exp^{\left(-q\Delta t/\tau_{\mathrm{ura}}\right)}+\left({\left(\sigma_{\mathrm{ml},\;i}^{\left(\ell \right)}\right)}^{2}+{\left(\sigma_{n,\;i}^{\ell )}\right)}^{2}\right)\exp^{\left(-q\Delta t/\tau_{\mathrm{ml}-n}\right)} \end{array}$$

where Δt = 1 s. *E* represents the expectation operation. $\sigma^{\left(\ell \right)}\left(i\right)$ is the standard deviation of ${\underline{\eta }}^{\left(\ell \right)}\left(i\right)$. β denotes the reciprocal of the time constant of an equivalent FOGM process for $\underline{\eta }\left(i\right)$. ${\left(\sigma^{\left(\ell \right)}\left(i\right)\right)}^{2}$ is

(18) $${\left(\sigma^{\left(\ell \right)}\left(i\right)\right)}^{2}={\left({\mathrm{mp}}^{\left(\ell \right)}\left(\theta^{\left(\ell \right)}\left(i\right)\right)\right)}^{2}\sigma_{\mathrm{tr}}^{2}+\sigma_{\mathrm{ura}}^{2}+({\left(\sigma_{\mathrm{ml},\;i}^{\left(\ell \right)}\right)}^{2}+\left(\sigma_{n,\;i}^{\left(\ell \right)}{)}^{2}\right)$$

Thus, based on the correlation between epochs *ℓ* and (ℓ+q), β (denoted as $\beta_{\ell q}$) is calculated as

(19) $$\begin{array}{c} \beta_{\ell q}=-\log\left(\frac{E\left({\underline{\eta }}^{\left(\ell \right)}\left(i\right){\underline{\eta }}^{\left(\ell +q\right)}\left(i\right)\right)}{{\left(\sigma^{\left(\ell \right)}\left(i\right)\right)}^{2}}\right)/\left(q\Delta t\right) \end{array}$$

It is evident from Equations (17) and (19) that $\beta_{\ell q}$ is a function of *q* and θ. It should be noted that ${\left(\sigma_{\mathrm{ml},\;i}^{\left(\ell \right)}{)}^{2}+(\sigma_{n,\;i}^{\left(\ell \right)}\right)}^{2}$ is also a function of θ.

Following the definition of Ψ for normalized innovations $\Delta {\underline{\tilde{\rho }}}^{*}$, $\Psi_{\mathrm{sub}}^{i}$ for satellite *i* mentioned after Equation (6) can be re-written as $\left[\begin{array}{ccccc} 1 & a_{11} & a_{12} & a_{13} & \cdots \\ a_{11} & 1 & a_{21} & a_{22} & \cdots \\ a_{12} & a_{21} & 1 & a_{31} & \cdots \\ & & \vdots & \end{array}\right]$, where $a_{\ell q}$ for an epoch *ℓ* with q=1,2,… is defined as

(20) $$a_{\ell q}=\frac{E\left({\underline{\eta }}^{\left(\ell \right)}\left(i\right){\underline{\eta }}^{\left(\ell +q\right)}\left(i\right)\right)}{\sigma^{\left(\ell \right)}\left(i\right)\sigma^{\left(\ell +q\right)}\left(i\right)}={\left(\exp^{\left(-\beta_{\ell q}\Delta t\right)}\right)}^{q}\frac{{\left(\sigma^{\left(\ell \right)}\left(i\right)\right)}^{2}}{\sigma^{\left(\ell \right)}\left(i\right)\sigma^{\left(\ell +q\right)}\left(i\right)}$$

$a_{\ell q}$ can be rearranged as

(21) $$a_{\ell q}=\left(\exp^{\left(-\beta_{\ell q}\Delta t\right)}\frac{\sigma^{\left(\ell \right)}\left(i\right)}{\sigma^{\left(\ell +1\right)}\left(i\right)}\right)\left(\exp^{\left(-\beta_{\ell q}\Delta t\right)}\frac{\sigma^{\left(\ell +1\right)}\left(i\right)}{\sigma^{\left(\ell +2\right)}\left(i\right)}\right)\cdots \left(\exp^{\left(-\beta_{\ell q}\Delta t\right)}\frac{\sigma^{\left(\ell +q-1\right)}\left(i\right)}{\sigma^{\left(\ell +q\right)}\left(i\right)}\right)$$

Representing the terms within the parentheses as $a_{\iota 1,\;\ell q}$, $a_{\ell q}$ = $\prod_{\iota =\ell }^{\iota =\ell +q-1}a_{\iota 1,\;\ell q}$. ℓq in the subscript of $a_{\iota 1,\;\ell q}$ denotes that β is obtained by considering the correlation between two epochs *ℓ* and ℓ+q. Maximum $a_{\iota 1,\;\ell q}$ over ι=ℓ,…,ℓ+q−1 is denoted as $a_{\iota 1,\;\ell q}^{\max}$, and the minimum $a_{\iota 1,\;\ell q}$ is $a_{\iota 1,\;\ell q}^{\min}$.

**Figure 1:**
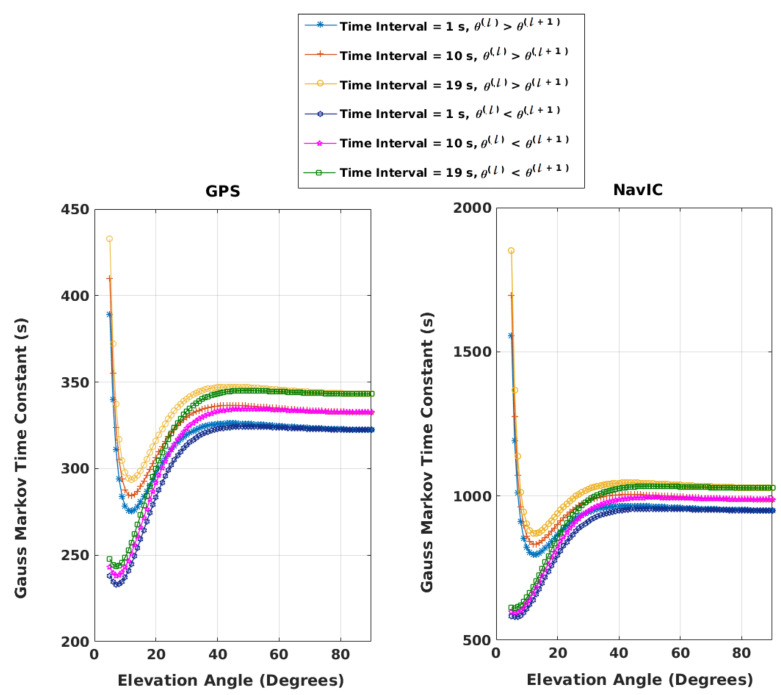
Equivalent time constants, $1/\beta_{\ell q}$ as a function of $\theta^{\left(\ell \right)}$; time interval = qΔt; Δt = 1 s.

**Figure 2:**
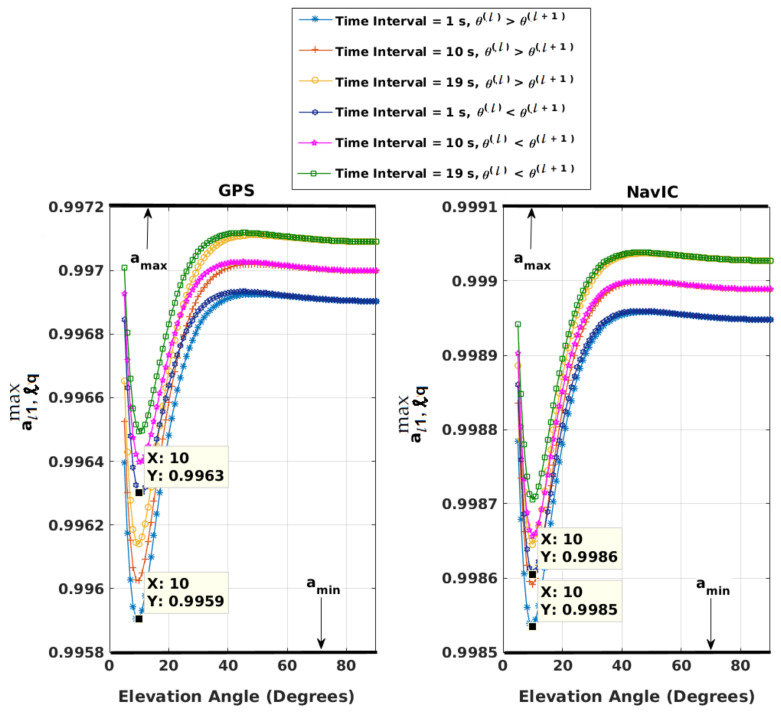
Variations of maximum $a_{\iota 1,\;\ell q}$ as a function of $\theta^{\left(\ell \right)}$; time interval = qΔt; Δt = 1 s.

$a_{\iota 1,\;\ell q}$s are calculated with $\beta_{\ell q}$ of Equation (19). Change in θ between two consecutive epochs, $\left(\theta^{\left(\iota +1\right)}\left(i\right)\;-\;\theta^{\left(\iota \right)}\left(i\right)\right)$, lies between $\pm \frac{v_{m}^{sat}+v_{m}^{user}}{r_{su}^{\left(\iota \right)}}\times \frac{180}{\pi }$, where $v_{m}^{sat}$ = maximum satellite speed, $v_{m}^{user}$ = maximum user speed = Mach 1, $r_{su}^{\left(\iota \right)}$ = range between the user and satellite at $\theta^{\left(\iota \right)}$ calculated using the approach of [36], and maximum user altitude = 15 km. With *M* and (N−M) less than or equal to 20, three different values of *q* are chosen for the calculation of $\beta_{\ell q}$. With maximum satellite and user speeds and user altitude, equivalent time constants, $1/\beta_{\ell q}$, and $a_{\iota 1,\;\ell q}^{\max}$ are plotted as a function of $\theta^{\left(\ell \right)}$ with increasing and decreasing θ over $t_{\ell },\ldots ,t_{\ell +q}$ and presented in Figure 1 and Figure 2, respectively, for both GPS and NavIC. $a_{\iota 1,\;\ell q}^{\min}$ is not shown to avoid clutter. It can be obtained along with the time constants and $a_{\iota 1,\;\ell q}^{\max}$ using the Matlab program matlab_program_figures_1_2.m provided in the Supplementary Materials. It is apparent that at a given $\theta^{\left(\ell \right)}$, the variation of $a_{\iota 1,\;\ell q}^{\max}$ with *q* is very small. The maximum difference is 0.0004 between *q* = 1 and *q* = 19. For $a_{\iota 1,\;\ell q}^{\min}$, it is 0.0002. The selected $a_{\max}\;\left(=\max_{q,\theta }a_{\iota 1,\;\ell q}^{\max}\right)$ and $a_{\min}\;\left(=\min_{q,\theta }a_{\iota 1,\;\ell q}^{\min}\right)$ are shown with thick black lines in Figure 2.

Exhaustive simulations are also performed to validate $a_{\min}$ and $a_{\max}$ of Figure 2. This is described next. Uniformly random user speeds within [$-v_{m}^{user}$$v_{m}^{user}$] are selected over *q* epochs. An initial satellite speed is chosen by a uniformly random sample within [$-v_{m}^{sat}$$v_{m}^{sat}$]. Then, the satellite speed is propagated over *q* epochs with an acceleration randomly picked between its minimum and maximum limits. This way, four hundred thousand sets of user and satellite speeds are obtained. Half of these are for GPS, and the other half are for NavIC. For GPS, with each $\theta^{\left(\ell \right)}$ between 5° and 90° and each of three *q*s, each of the two hundred thousand sets of speeds is used to calculate $\theta^{\left(\ell +1\right)},\ldots ,\theta^{\left(\ell +q\right)}$ for a given user altitude, and the corresponding $a_{\iota 1,\;\ell q}^{\min}$ and $a_{\iota 1,\;\ell q}^{\max}$ are noted. With half of the speeds, $\theta^{\left(\iota +1\right)}=\theta^{\left(\iota \right)}\;+\;\frac{v_{sat}^{\left(\iota \right)}+v_{user}^{{}^{(}\iota )}}{r_{su}^{\left(\iota \right)}}\times \frac{180}{\pi },\;\iota =\ell ,\ldots \ell +q-1$, where $v_{sat}^{\left(\iota \right)}$ and $v_{user}^{\left(\iota \right)}$ are satellite and user speeds at epoch ι, and $\theta^{\left(\iota +1\right)}$ = $\theta^{\left(\iota \right)}\;-\;\frac{v_{sat}^{\left(\iota \right)}+v_{user}^{\left(\iota \right)}}{r_{su}^{\left(\iota \right)}}\times \frac{180}{\pi }$ with the other one hundred thousand sets. Considering all sets of data, $\left(\max_{q,\theta }a_{\iota 1,\;\ell q}^{\max}\right)$ is found to be less than the selected $a_{\max}$ and $\left(\min_{q,\theta }a_{\iota 1,\;\ell q}^{\min}\right)\;>\;a_{\min}$. The same is repeated for NavIC with the other two hundred thousand sets. This is performed numerous times with the Matlab program matlab_program_figures_1_2.m of the Supplementary Materials. Next, it is repeated for each user altitude from 0 to 15 km in steps of 5 km. Finally, in each simulation, the user altitude is varied randomly with uniform distribution between 0 and 15 km over *q* epochs. In all simulations, $a_{\max}$ and $a_{\min}$ of Figure 2 are found to be the correct upper and lower bounds.

The structure of the corresponding $\Psi_{\mathrm{sub},\;\max}^{i}$ remains the same. That is, $\Psi_{\mathrm{sub},\;\max=}^{i}\left[\begin{array}{ccccc} 1 & a_{\max} & a_{\max}^{2} & a_{\max}^{3} & \cdots \\ a_{\max} & 1 & a_{\max} & a_{\max}^{2} & \cdots \\ a_{\max}^{2} & a_{\max} & 1 & a_{\max} & \cdots \\ & & \vdots & \end{array}\right]$.

Next, modifications of Ψ due to uncertainties in the knowledge of actual $a_{\ell q}$ and initial artificial white noise are provided.

## 4. Ψ with Uncertain Parameters and White Noise

In this section, Ψ (or $\Psi_{\mathrm{sub}}^{i}$) is first appropriately modeled with underlying parameters within a range to include the effect of uncertainties in their knowledge, and to account for initial artificial white noise. Subsequently, with the help of extensive simulations, it is shown that $\Psi_{\max}$ can be used in place of Ψ to form test statistics and PLs.

### 4.1. Uncertain $a_{\ell q}$

Uncertainties in the knowledge of $a_{\ell q}$ arise because actual values of the parameters needed to calculate it are not known. Suppose the actual value of $\tau_{\mathrm{tr}}$ for a satellite is $\varrho_{\mathrm{tr}}$ times 25 h (modeled value), where $\varrho_{\mathrm{tr},\;\min}\;\leq \;\varrho_{\mathrm{tr}}\;\leq \;1$. Similarly, the scale factor for $\tau_{\mathrm{ura}}$ is $\varrho_{\mathrm{ura}}$, which lies between $\varrho_{\mathrm{ura},\;\min}$ and one. Actual $\sigma_{\mathrm{tr}}^{2}$, $\sigma_{\mathrm{ura}}^{2}$ and $\left(\sigma_{\mathrm{ml}}^{2}+\sigma_{n}^{2}\right)$ also take on values below their respective modeled values. Their actual values can be obtained by multiplying scale factors $K_{\mathrm{tr}}$, $K_{\mathrm{ura}}$, and $K_{\mathrm{ml}-n}$, respectively, to the respective modeled values. The lower limits of these scale factors are $K_{\mathrm{tr},\;\min}$, $K_{\mathrm{ura},\;\min}$, and $K_{\mathrm{ml}-n,\;\min}$, respectively, and the upper limits are one. All lower limits should be determined by prior analysis. A possible approach would be to analyze several months of data, following [32,33] for the tropospheric error and URA, respectively. The autocorrelation functions, power spectral densities, and error distributions of the data can be formed to determine both upper and lower bounds of the time constants and variances. The ratios of the lower to upper bounds will give the lower limits of the scale factors, and the upper bounds will be the modeled values discussed earlier. Although not considered in this paper, modeled values may change across satellites. Similar analyses may be done for multipath and receiver noise, but this will be application specific. Equation (17) is now re-written as

(22) $$\begin{array}{cc} E\left({\underline{\eta }}^{\left(\ell \right)}\left(i\right){\underline{\eta }}^{\left(\ell +q\right)}\left(i\right)\right) & ={\left(\sigma^{\left(\ell \right)}\left(i\right)\right)}^{2}\exp^{\left(-\beta q\Delta t\right)}={\mathrm{mp}}^{\left(\ell \right)}\left(\theta^{\left(\ell \right)}\left(i\right)\right){\mathrm{mp}}^{\left(\ell +q\right)}\left(\theta^{\left(\ell +q\right)}\left(i\right)\right)K_{\mathrm{tr}}\sigma_{\mathrm{tr}}^{2}\exp^{\left(-q\Delta t/\left(\varrho_{\mathrm{tr}}\tau_{\mathrm{tr}}\right)\right)}+\\ \; & K_{\mathrm{ura}}\sigma_{\mathrm{ura}}^{2}\exp^{\left(-q\Delta t/\left(\varrho_{\mathrm{ura}}\tau_{\mathrm{ura}}\right)\right)}+K_{\mathrm{ml}-n}\left({\left(\sigma_{\mathrm{ml},\;i}^{\left(\ell \right)}\right)}^{2}+{\left(\sigma_{n,\;i}^{\ell )}\right)}^{2}\right)\exp^{\left(-q\Delta t/\tau_{\mathrm{ml}-n}\right)} \end{array}$$

In this work, no uncertainty in the time constant of multipath and noise is assumed. However, this can be relaxed if needed. Using Equation (22), modified $\beta_{\ell q}$, $\beta_{\ell q}^{\mathrm{mod}}$ is

(23) $$\beta_{\ell q}^{\mathrm{mod}}=-\log\left(\frac{E\left({\underline{\eta }}^{\left(\ell \right)}\left(i\right){\underline{\eta }}^{\left(\ell +q\right)}\left(i\right)\right)}{{\left(\sigma^{\left(\ell \right)}\left(i\right)\right)}^{2}}\right)/\left(q\Delta t\right)$$

Comparing Equations (17) and (22), it is evident that $\exp^{\left(-\beta_{\ell q}^{\mathrm{mod}}q\Delta t\right)}$≤$\exp^{\left(-\beta_{\ell q}q\Delta t\right)}$. The actual $\sigma^{\left(\ell \right)}{\left(i\right))}^{2}$, $\sigma_{\mathrm{act}}^{\left(\ell \right)}{\left(i\right))}^{2}$ is given by

(24) $$\sigma_{\mathrm{act}}^{\left(\ell \right)}{\left(i\right))}^{2}={\left({\mathrm{mp}}^{\left(\ell \right)}\left(\theta^{\left(\ell \right)}\left(i\right)\right)\right)}^{2}K_{\mathrm{tr}}\sigma_{\mathrm{tr}}^{2}+K_{\mathrm{ura}}\sigma_{\mathrm{ura}}^{2}+K_{\mathrm{ml}-n}\left({\left(\sigma_{\mathrm{ml},\;i}^{\left(\ell \right)}\right)}^{2}+{\left(\sigma_{n,\;i}^{\ell )}\right)}^{2}\right)$$

As Equation (20) has the modeled values of σ in the denominator, it reduces to

(25) $$a_{\ell q}={\left(\exp^{\left(-\beta_{\ell q}^{\mathrm{mod}}\Delta t\right)}\right)}^{q}\frac{{\left(\sigma^{\left(\ell \right)}\left(i\right)\right)}^{2}}{\sigma^{\left(\ell \right)}\left(i\right)\sigma^{\left(\ell +q\right)}\left(i\right)}$$

With the rearrangement of terms as before,

(26) $$a_{\ell q}=\left(\exp^{\left(-\beta_{\ell q}^{\mathrm{mod}}\Delta t\right)}\frac{\sigma^{\left(\ell \right)}\left(i\right)}{\sigma^{\left(\ell +1\right)}\left(i\right)}\right)\cdots \left(\exp^{\left(-\beta_{\ell q}^{\mathrm{mod}}\Delta t\right)}\frac{\sigma^{\left(\ell +q-1\right)}\left(i\right)}{\sigma^{\left(\ell +q\right)}\left(i\right)}\right)$$

The *ℓ*th diagonal term of $\Psi_{\mathrm{sub}}^{i}$ is

(27) $$a_{\ell 0}={\left(\frac{\sigma_{\mathrm{act}}^{\left(\ell \right)}\left(i\right)}{\sigma^{\left(\ell \right)}\left(i\right)}\right)}^{2}$$

It should be noted that the term $\left(\sigma_{\mathrm{act}}^{\left(\ell \right)}\left(i\right)/\sigma^{\left(\ell \right)}\left(i\right)\right)$ is less than or equal to one as $\sigma^{\left(\ell \right)}\left(i\right)$≥$\sigma_{\mathrm{act}}^{\left(\ell \right)}\left(i\right)$. If $K_{\min}^{\prime }$ is the minimum of $K_{\mathrm{tr},\;\min}$, $K_{\mathrm{ura},\;\min}$, and $K_{\mathrm{ml}-n,\;\min}$, then using Equation (24), $K_{\min}^{\prime }$ ≤ $a_{\ell 0}$ ≤ 1.

Multiplying and dividing ${\left(\exp^{\left(-\beta_{\ell q}\Delta t\right)}\right)}^{q}$ in Equation (26) and rearranging terms,

(28) $$a_{\ell q}=\left(\exp^{\left(-\beta_{\ell q}\Delta t\right)}\frac{\sigma^{\left(\ell \right)}\left(i\right)}{\sigma^{\left(\ell +1\right)}\left(i\right)}\right)\cdots \left(\exp^{\left(-\beta_{\ell q}\Delta t\right)}\frac{\sigma^{\left(\ell +q-1\right)}\left(i\right)}{\sigma^{\left(\ell +q\right)}\left(i\right)}\right){\left(\frac{\exp^{\left(-\beta_{\ell q}^{\mathrm{mod}}\Delta t\right)}}{\exp^{\left(-\beta_{\ell q}\Delta t\right)}}\right)}^{q}$$

Using $a_{\min}$ and $a_{\max}$, and $\exp^{\left(-\beta_{\ell q}^{\mathrm{mod}}q\Delta t\right)}$≤$\exp^{\left(-\beta_{\ell q}q\Delta t\right)}$ noted after Equation (23),

(29) $$a_{\min}^{q}\left(\min_{q,\;\theta \;K,\;\varrho }{\left(\frac{\exp^{\left(-\beta_{\ell q}^{\mathrm{mod}}\Delta t\right)}}{\exp^{\left(-\beta_{\ell q}\Delta t\right)}}\right)}^{q}\right)\leq a_{\ell q}\leq a_{\max}^{q}$$

where $\min_{q,\;\theta \;K,\;\varrho }{\left(\exp^{\left(-\beta_{\ell q}^{\mathrm{mod}}\Delta t\right)}/\exp^{\left(-\beta_{\ell q}\Delta t\right)}\right)}^{q}$ is the minimum value obtained over all values of K, ϱ, θ, and *q* (maximum *q* is (M−1) for $\alpha_{1}^{\prime }$ and (N−M−1) for $\alpha_{2}^{\prime }$). For a given *q* and θ, ${\left(\exp^{\left(-\beta_{\ell q}^{\mathrm{mod}}\Delta t\right)}/\exp^{\left(-\beta_{\ell q}\Delta t\right)}\right)}^{q}$ is minimum when all Ks and ϱs are set to their respective minimum values (see Equations (17), (19), (22), and (23)). This is greater than or equal to the value $K_{\min}^{\prime \prime }$ obtained by setting all Ks to $K_{\min}^{\prime }$. $K_{\min}^{\prime \prime }$ is close but less than $K_{\min}^{\prime }$. The minimum of all $K_{\min}^{\prime \prime }$s over all values of θ and *q* is set as the lower bound, and denoted as $K_{\min}$ (0 < $K_{\min}$ < 1). It can be determined by following the instructions in matlab_program_figures_1_2.m and then running it. Next, a provision is made to include artificial white noise in $a_{\ell q}$.

### 4.2. White Noise in $a_{\ell q}$

White noise is injected initially in the carrier-smoothed pseudorange measurement error model of the SKF to deal with transient errors in position estimates. During this initial period, the modeled $\sigma^{\left(\ell \right)}\left(i\right)$ is given by

(30) $${\left(\sigma_{\mathrm{init}}^{\left(\ell \right)}\left(i\right)\right)}^{2}={\left(\sigma^{\left(\ell \right)}\left(i\right)\right)}^{2}+{\left(\sigma_{\mathrm{wh}}^{\left(\ell \right)}\left(i\right)\right)}^{2}$$

If the injected white noise variance, ${\left(\sigma_{\mathrm{wh}}^{\left(\ell \right)}\left(i\right)\right)}^{2}$, is $K_{\mathrm{wh}}\sigma^{\left(\ell \right)}{\left(i\right))}^{2}$, ${\left(\sigma_{\mathrm{init}}^{\left(\ell \right)}\left(i\right)\right)}^{2}$ becomes equal to $\left(K_{\mathrm{wh}}+1\right){\left(\sigma^{\left(\ell \right)}\left(i\right)\right)}^{2}$. $K_{\mathrm{wh}}$ is a known scale factor as white noise is artificially included. With this, Equation (26) is changed to

(31) $$a_{\ell q}=\left(\exp^{\left(-\beta_{\ell q}^{\mathrm{mod}}\Delta t\right)}\frac{\sigma^{\left(\ell \right)}\left(i\right)}{\sigma^{\left(\ell +1\right)}\left(i\right)}\right)\cdots \left(\exp^{\left(-\beta_{\ell q}^{\mathrm{mod}}\Delta t\right)}\frac{\sigma^{\left(\ell +q-1\right)}\left(i\right)}{\sigma^{\left(\ell +q\right)}\left(i\right)}\right)\left(\frac{\sigma^{\left(\ell \right)}\left(i\right)}{\sigma_{\mathrm{init}}^{\left(\ell \right)}\left(i\right)}\right)\left(\frac{\sigma^{\left(\ell +q\right)}\left(i\right)}{\sigma_{\mathrm{init}}^{\left(\ell +q\right)}\left(i\right)}\right)$$

Replacing the last two terms on the right,

(32) $$a_{\ell q}=\left(\exp^{\left(-\beta_{\ell q}^{\mathrm{mod}}\Delta t\right)}\frac{\sigma^{\left(\ell \right)}\left(i\right)}{\sigma^{\left(\ell +1\right)}\left(i\right)}\right)\cdots \left(\exp^{\left(-\beta_{\ell q}^{\mathrm{mod}}\Delta t\right)}\frac{\sigma^{\left(\ell +q-1\right)}\left(i\right)}{\sigma^{\left(\ell +q\right)}\left(i\right)}\right)\frac{1}{\left(K_{\mathrm{wh}}+1\right)}$$

Similarly, the *ℓ*th diagonal term of $\Psi_{\mathrm{sub}}^{i}$ is given by

(33) $$a_{\ell 0}={\left(\frac{\sigma_{\mathrm{act}}^{\left(\ell \right)}\left(i\right)}{\sigma^{\left(\ell \right)}\left(i\right)}\right)}^{2}\frac{1}{\left(K_{\mathrm{wh}}+1\right)}\leq 1$$

Its lower bound is $K_{\min}^{\prime }/\left(K_{\mathrm{wh}}+1\right)$. Using Equation (29), $a_{\ell q}$ is bounded by

(34) $$a_{\min}^{q}\frac{K_{\min}}{\left(K_{\mathrm{wh}}+1\right)}\leq a_{\ell q}\leq a_{\max}^{q}$$

### 4.3. Simulation Results

Having modeled Ψ with underlying parameters residing within a range, results of extensive simulations are shown to justify that $\Psi_{\max}$ can be used in place of Ψ to determine test statistics and PLs. In other words, it is illustrated that $\lambda_{\Theta ,\;2}^{\max}$ or $\lambda_{\Omega }^{\max}$ with Θ formed as ${\left(cI+\Psi_{\max}\right)}^{-1}$ is less than or equal to one (see after Equation (5)). In addition, $\lambda_{\Theta ,\;2}^{\min}$ or $\lambda_{\Omega }^{\min}$ is shown to be more than the minimum eigenvalue of ${\tilde{S}}^{\prime T}\Psi_{\max}{\tilde{S}}^{\prime }{\tilde{S}}^{\prime T}\Theta {\tilde{S}}^{\prime }$ (=$\Omega_{1}$) times a known scale factor. This is needed to ensure that the calculated Γ of Equation (11) is larger than its actual value (see Equation (10) and the paragraph after Equation (11) for justification of this). The actual value of Γ would need the actual Ψ, which is not known.

**Figure 3:**
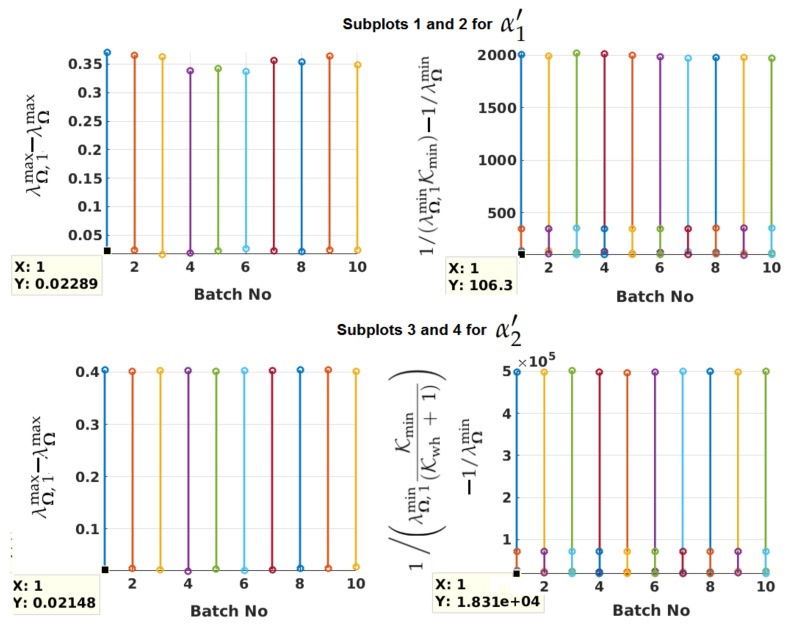
Differences between maximum (and minimum) eigenvalues of $\Omega_{1}$ and Ω over five hundred thousand simulations for each $\alpha_{1}^{\prime }$ and $\alpha_{2}^{\prime }$.

For the simulation results of this section, lower limits of the scale factors used to account for uncertain parameters are: $\varrho_{\mathrm{tr},\;\min}$ = 0.5; $\varrho_{\mathrm{ura},\;\min}$ = 0.5; $K_{\mathrm{tr},\;\min}$ = 0.25; $K_{\mathrm{ura},\;\min}$ = 0.25; and $K_{\mathrm{ml}-n,\;\min}$ = 0.25. For the selected lower limits, $K_{\min}$ can be obtained a priori by running matlab_program_figures_1_2.m. $K_{\mathrm{wh}}$ is 5 for white noise overbound. Scale factors are uniformly distributed between their lower and upper limits. While they are changed between simulations, for a given satellite they are assumed to be constant over *M* epochs (for $\alpha_{1}^{\prime }$) or (N−M) epochs (for $\alpha_{2}^{\prime }$). *M* = 10, *N* = 20, and the time interval between two epochs Δt = 1 s. Fourteen GPS and seven NavIC satellites are considered. A brief description of the algorithm needed for simulations is provided in Appendix A. Matlab implementation of the algorithm can be found in the Supplementary Materials (matlab_program_algorithm_1_ts1_figure_3.m and matlab_program_algorithm_1_ts2_figure_3.m), where all inputs can be changed as needed, and results with the changed values can be generated.

The algorithm also has a provision to remove a satellite at the *M*th or (N−M)th epoch to check the change in visibility and remove white noise injection from a random intermediate time epoch. Satellites are excluded when the elevation angle is below the 5° mask angle. A satellite can be chosen to come into visibility after a random number of epochs.

LOS vectors of H are randomly selected without considering actual GPS and NavIC satellite orbits. Hence, results of these simulations are likely to hold for other constellations if error model parameters, frequency bands, and $a_{\min}$ and $a_{\max}$ are suitably changed. Although there is an inherent relationship between elevation angles and H, it is not important here as long as the structure of the simulated H resembles that of the actual measurement model matrix. That is, the LOS vector constraint of each row is satisfied, the clock bias columns are properly formed, and the rank of H is 5. With this, valid measurement model matrices would just be a subset of the wide range of H matrices considered in the simulations.

Figure 3 depicts the difference between the maximum eigenvalues of $\Omega_{1}$ and Ω for both $\alpha_{1}^{\prime }$ and $\alpha_{2}^{\prime }$ when θ decreases with time (see subplots 1 and 3—one below the other on the left). $\lambda_{\Omega ,\;1}^{\min}$ and $\lambda_{\Omega ,\;1}^{\max}$ are the minimum and maximum eigenvalues of $\Omega_{1}$, respectively. In each simulation, the initial θ of a satellite is chosen with a uniform random variable between 5.1° and 90°. Satellite speed is constant at its maximum value. Five hundred thousand simulations are divided into ten batches for ease of plotting in Matlab, with each batch having 50,000 runs. The minimum and maximum differences over 50,000 simulations in a batch are shown as the end points of a vertical line. Within a batch, 10,000 runs have white noise injected up to some random intermediate epochs. Since the minimum difference between $\lambda_{\Omega ,\;1}^{\max}$ and $\lambda_{\Omega }^{\max}$ is greater than zero in all batches, $\lambda_{\Omega }^{\max}$ is less than $\lambda_{\Omega ,\;1}^{\max}$, which in turn is less than or equal to one by proper calculation of *c*. The differences between $1/(\lambda_{\Omega ,\;1}^{\min}K_{\min})$ and $1/\lambda_{\Omega }^{\min}$ for $\alpha_{1}^{\prime }$ (and between $1/(\lambda_{\Omega ,\;1}^{\min}\frac{K_{\min}}{\left(K_{\mathrm{wh}}\;+\;1\right)})$ and $1/\lambda_{\Omega }^{\min}$ for $\alpha_{2}^{\prime }$) are illustrated in the second (and fourth) subplot of the same figure. In this case, the longer vertical line of each batch belongs to the 10,000 simulations with artificial white noise. It is evident that $1/\lambda_{\Omega }^{\min}$ < $1/(\lambda_{\Omega ,\;1}^{\min}K_{\min})$ for $\alpha_{1}^{\prime }$, and $1/\lambda_{\Omega }^{\min}$ < $1/(\lambda_{\Omega ,\;1}^{\min}\frac{K_{\min}}{\left(K_{\mathrm{wh}}\;+\;1\right)})$ for $\alpha_{2}^{\prime }$. $\lambda_{\Omega }^{\min}$ is verified to be always positive. It can also be concluded that $\sqrt{\lambda_{\Omega }^{\max}}/\lambda_{\Omega }^{\min}$ < $\sqrt{\lambda_{\Omega ,\;1}^{\max}}/\left(\lambda_{\Omega ,\;1}^{\min}K_{\min}\right)$ for $\alpha_{1}^{\prime }$. Similarly, for $\alpha_{2}^{\prime }$, $\sqrt{\lambda_{\Omega }^{\max}}/\lambda_{\Omega }^{\min}$ < $\sqrt{\lambda_{\Omega ,\;1}^{\max}}/\left(\lambda_{\Omega ,\;1}^{\min}\frac{K_{\min}}{\left(K_{\mathrm{wh}}\;+\;1\right)}\right)$. Hence, using $\Psi_{\max}$ in place of Ψ, $\alpha_{1}^{\prime }$, $\alpha_{2}^{\prime }$ and PLs can be calculated. Although not shown, the same conclusion can be derived with increasing elevation angle and changing satellite speeds, and verified with the provided Matlab programs.

It should be noted that when *c* is adaptively calculated for $\alpha_{1}^{\prime }$, $\lambda_{\Omega }^{\max}$ is less than $\lambda_{\Omega ,\;1}^{\max}$ and one only if *c* is more than or equal to a minimum value, $c_{\min}$. It is found that $c_{\min}$ is a function of *M* and elevation angle. Appendix B explains how $c_{\min}$ is obtained. During adaptive calculation, the initial guess of *c* is taken as $c_{\min}$ or higher. Next, a tighter value of Γ is determined. The effect of $W_{rr,\;\ell }$ overbounding the actual pseudorange rate (or precise range rate) error covariance is also discussed.

## 5. Determination of Γ

Substituting Equation (5) in the first term of Equation (6) on the right and having a similar representation for the second term, one can write

(35) $$\alpha_{j}^{2}={{\underline{\eta }}_{r}}^{T}\Theta_{2}{\underline{\eta }}_{r}+{{\underline{\eta }}_{rr}}^{T}\Theta_{5}{\underline{\eta }}_{rr}$$

where ${\underline{\eta }}_{rr}$ has unit-variance independent elements. $\Theta_{5}$ would be the identity matrix only if $W_{rr,\;\ell }$ of Equation (6) is equal to the actual pseudorange rate error covariance matrix. However, $W_{rr,\;\ell }$ generally overbounds the actual error covariance. As a result, the maximum eigenvalue of $\Theta_{5}$ is less than or equal to one, and the minimum eigenvalue is at some value greater than zero [29]. They are denoted as $\lambda_{\Theta ,\;5}^{\max}$ and $\lambda_{\Theta ,\;5}^{\min}$, respectively, and determined using prior knowledge of overbounding different satellite range rate errors. $\tilde{\Theta }$ of Equation (11) thus changes to

(36) $$\tilde{\Theta }=\left[\begin{array}{cc} \Theta_{2} & \left[0\right]\\ \left[0\right] & \Theta_{5} \end{array}\right]$$

It is apparent that $\lambda_{\tilde{\Theta }}^{\max}$ = $\max\left\{\lambda_{\Theta ,\;2}^{\max},\;\lambda_{\Theta ,\;5}^{\max}\right\}$ and $\lambda_{\tilde{\Theta }}^{\min}$ = $\min\left\{\lambda_{\Theta ,\;2}^{\min},\;\lambda_{\Theta ,\;5}^{\min}\right\}$. The upper and lower bounds of $\lambda_{\Theta ,\;2}^{\max}$ and $\lambda_{\Theta ,\;2}^{\min}$, respectively, as determined in the previous section, are used in place of their actual, unknown values.

With the formulation of $\tilde{\Theta }$ after Equation (11), where $\lambda_{\tilde{\Theta }}^{\max}$ = 1, Γ can be directly obtained from the lookup table. As Γ was shown to increase with decreasing $\lambda_{\tilde{\Theta }}^{\min}$, the next entry of the lookup table immediately higher than the calculated $1/\lambda_{\tilde{\Theta }}^{\min}$ is considered. With $\tilde{\Theta }$ of Equation (36), and the minimum and maximum eigenvalues defined after it, Γ is now calculated in the following way. In this case, $\lambda_{\tilde{\Theta }}^{\max}$ is not necessarily equal to one. First, Γ is obtained from the lookup table the same way as before, using $P_{MD,\;i}^{alloc}$, DOF, and the next ratio entry higher than the calculated $\sqrt{\lambda_{\tilde{\Theta }}^{\max}}/\lambda_{\tilde{\Theta }}^{\min}$. This Γ is not the right one when $\lambda_{\tilde{\Theta }}^{\max}$≠ 1, and therefore is denoted by $\Gamma^{\prime }$. In addition, for the lookup table, the detection threshold, $T_{th,\;1}$, is now determined from the non-central chi-squared distribution with the required DOF and some predetermined natural bias [37]. Using $\Gamma^{\prime }$, the $\Gamma^{*}$ of Equation (11) is calculated as

(37) $$\sqrt{\Gamma^{*}}=\sqrt{\Gamma^{\prime }}-T_{th,\;1}+T_{th,\;1}{\left(\frac{\sqrt{\lambda_{\tilde{\Theta }}^{\max}}}{\lambda_{\tilde{\Theta }}^{\min}}\right)}_{\mathrm{lookup}}$$

where ${\left(\frac{\sqrt{\lambda_{\tilde{\Theta }}^{\max}}}{\lambda_{\tilde{\Theta }}^{\min}}\right)}_{\mathrm{lookup}}$ is the lookup table entry. Using Equation (11), the correct Γ is given as

(38) $$\sqrt{\Gamma }=\left(\sqrt{\Gamma^{*}}-T_{th,\;1}{\left(\frac{\sqrt{\lambda_{\tilde{\Theta }}^{\max}}}{\lambda_{\tilde{\Theta }}^{\min}}\right)}_{\mathrm{lookup}}\right)\sqrt{\lambda_{\tilde{\Theta }}^{\max}}+T_{th,\;1}$$

Substituting Equation (37) in the preceding equation

(39) $$\sqrt{\Gamma }=\left(\sqrt{\Gamma^{\prime }}-T_{th,\;1}\right)\sqrt{\lambda_{\tilde{\Theta }}^{\max}}+T_{th,\;1}$$

If $\sqrt{\Gamma^{\prime }}$<$T_{th,\;1}$, $\sqrt{\Gamma^{\prime }}$ is taken as $T_{th,\;1}$, which may occur at a very large DOF. As ${\left(\frac{\sqrt{\lambda_{\tilde{\Theta }}^{\max}}}{\lambda_{\tilde{\Theta }}^{\min}}\right)}_{\mathrm{lookup}}$ > calculated $\left(\frac{\sqrt{\lambda_{\tilde{\Theta }}^{\max}}}{\lambda_{\tilde{\Theta }}^{\min}}\right)$, $\Gamma^{\prime }$ is greater than the actual $\Gamma^{\prime }$. Hence, the Γ from Equation (39) is more than the actual Γ. The Γ corresponding to $\alpha_{2}$ is also obtained in the same way.

Performance and salient features of the modified KF RAIM are studied next for simulated scenarios of aircraft flight.

## 6. Simulation Studies

**Figure 4:**
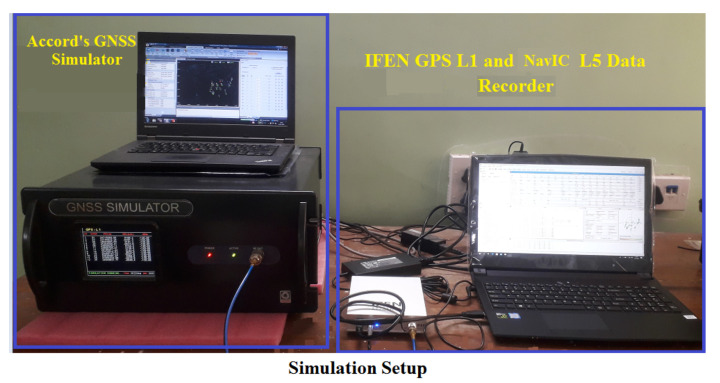
GNSS signal simulation and data collection setup.

**Figure 5:**
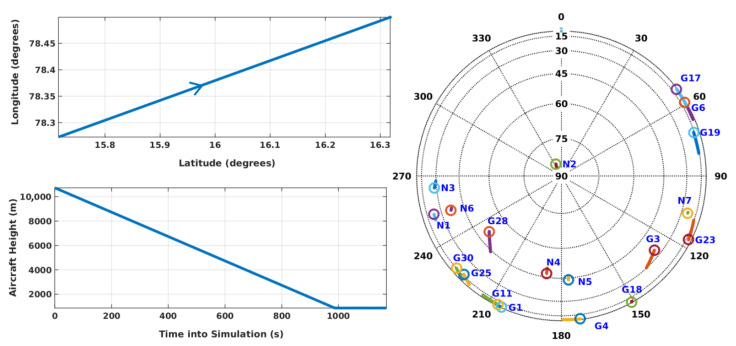
Simulated aircraft trajectory and skyplot of visible GPS and NavIC satellites. Aircraft height is shown above the WGS84 reference ellipsoid. In the skyplot, the circle marks the final position of a satellite in the trace. ‘G’ is for GPS, and ‘N’ is for NavIC.

In this section, various aspects of KF RAIM are analyzed using simulated GPS L1 and NavIC L5 signals for aircraft flight. For this purpose, a GNSS simulator from Accord Software and Systems is used [38]. It has a provision to load GPS and NavIC Yuma files, and configure user motion profiles and required error models (e.g., ionospheric and tropospheric errors). In the simulation, the ionospheric error is modeled by the Klobuchar model and the tropospheric error by RTCA98 [31]. Simulated signals are received by an IFEN receiver front end and digitized at 20 MHz. Stored 20 MHz IF data are processed by a developed GPS L1-NavIC L5 software receiver. Figure 4 shows the signal simulation and data collection setup. Performance is first analyzed in detail for a segment of an aircraft flight path during descent and approach, which is illustrated in Figure 5 along with GPS-NavIC satellite visibility.

**Figure 6:**
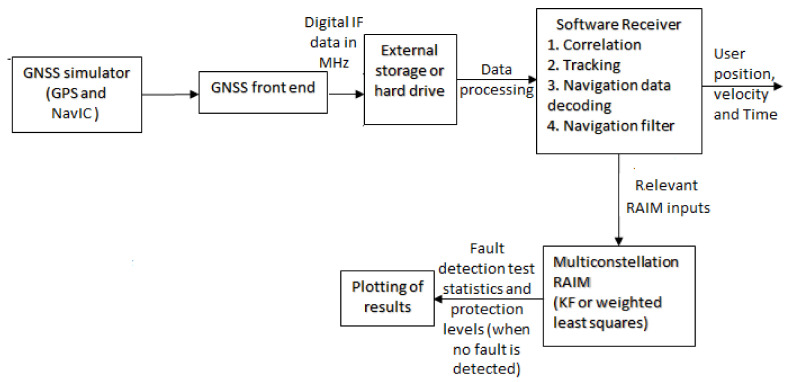
IF data processing block diagram.

The IF data processing block diagram is depicted in Figure 6. A GPS L1-NavIC L5 software radio is developed in Matlab to perform all receiver operations. Following acquisition, it implements tracking of the visible satellites in parallel processing mode to speed up computation time. Tracking outputs are then sent to the navigation filter to estimate user position, velocity, and time. Relevant RAIM inputs are passed from the filter to the multi-constellation KF or WLS RAIM algorithm to carry out fault detection and PL computation.

The receiver removes the ionospheric error using the Klobuchar model but uses a different model (modified Hopfield [39]) for the tropospheric error. The residual zenith tropospheric error is represented by an FOGM process in the navigation filter with a zero mean, standard deviation of 0.2 m, and time constant of 25 h. It bounds the actual error in the simulated measurements. There is no residual ionospheric error. Satellite orbit and clock error and multipath were not included in the simulation. They are, however introduced in the carrier-smoothed pseudorange measurements following the respective FOGM models. As a result, with each run, different positioning error results can be obtained. Range rate measurements, on the other hand, are generated from the Doppler frequencies of the carrier phase tracking loops. Only white noise with standard deviations obtained from the tracking loops of the receiver is modeled for range rate measurements. $K_{\min}$ for KF RAIM is taken as 0.75${}^{2}$ to be over conservative (see after Equation (34)).

**Figure 7:**
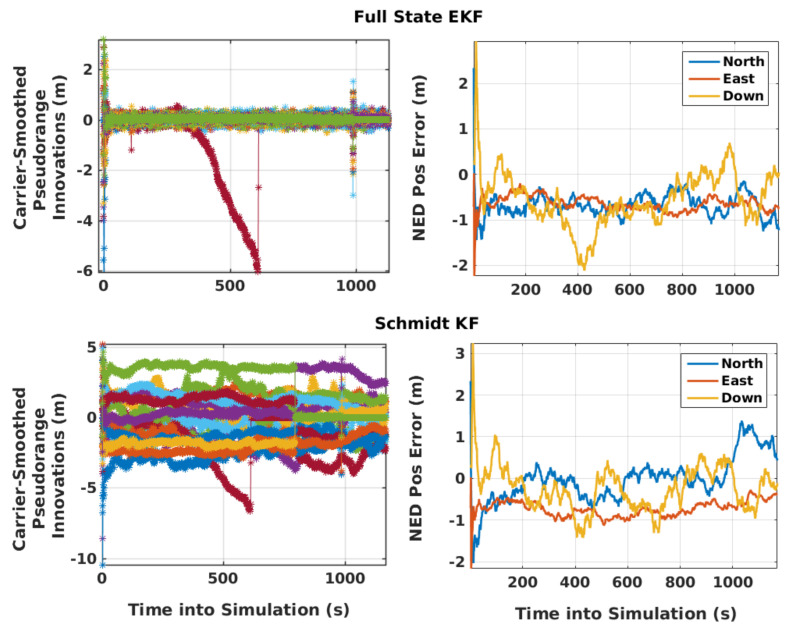
EKF and SKF carrier smoothed-pseudorange measurement innovations and position estimation errors in the NED frame. The ramp error in innovations is due to a low elevation satellite below the mask angle, which is not considered for estimation.

There is a provision for three different navigation filters in the receiver: WLS, SKF, and full-state EKF. WLS estimates error in the a priori ECEF position and velocity of the aircraft, and the receiver clock offsets and drifts for GPS and NavIC from respective truths. It calculates the measurement error standard deviation considering the residual tropospheric error, URA, multipath, and noise. Full-state EKF and SKF estimate error in the a priori acceleration states of the aircraft in ECEF apart from those mentioned for WLS. In addition, the full-state EKF estimates three states for the measurement error of each visible satellite (residual zenith tropospheric error, satellite clock and orbit error, and multipath and noise) using the corresponding FOGM models. The measurement update interval is 1 s. EKF and SKF position estimation errors in the NED frame and carrier-smoothed pseudorange measurement innovations are shown in Figure 7 for a simulation run. As the SKF treats residual errors, multipath, and noise as “consider parameters”, its innovations remain biased, as opposed to those of full-state EKF.

### 6.1. Analyses of KF RAIM

In this paper, the following parameters are considered for analyses of KF RAIM. The continuity budget for false alert, $P_{FA}$ is $3.33\times 10^{-7}$/sample [31]. The allocated probability of hazardously misleading information (HMI), $P_{HMI}^{alloc}$ is $10^{-7}$/h during aircraft descent and initial and intermediate approach, and $10^{-7}$/approach during the final approach [40]. The prior probability of narrow fault of GPS and NavIC satellites is $10^{-4}$/satellite/h. Assuming a one-hour mean time to notify by the constellation service provider, the prior probability of fault is $10^{-4}$/satellite as the simulation duration is less than an hour [41]. Constellation-wide faults are not taken into account. $P_{HMI}^{alloc}$ for the horizontal position, $P_{HMI,\;H}^{alloc}$, is 10% of $P_{HMI}^{alloc}$. $P_{HMI}^{alloc}$ for the vertical position, $P_{HMI,\;V}^{alloc}$, is the remaining 90%. For the prior fault probability and allocated integrity budget, at most two simultaneous independent satellite failures need to be monitored, as justified in [27]. The probability of three or more non-monitored faults $P_{nm}$, being less than the $P_{HMI}^{alloc}$, is subtracted from the latter. During the final approach phase, vertical and horizontal alert limits are considered to be 50 m and 40 m.

**Figure 8:**
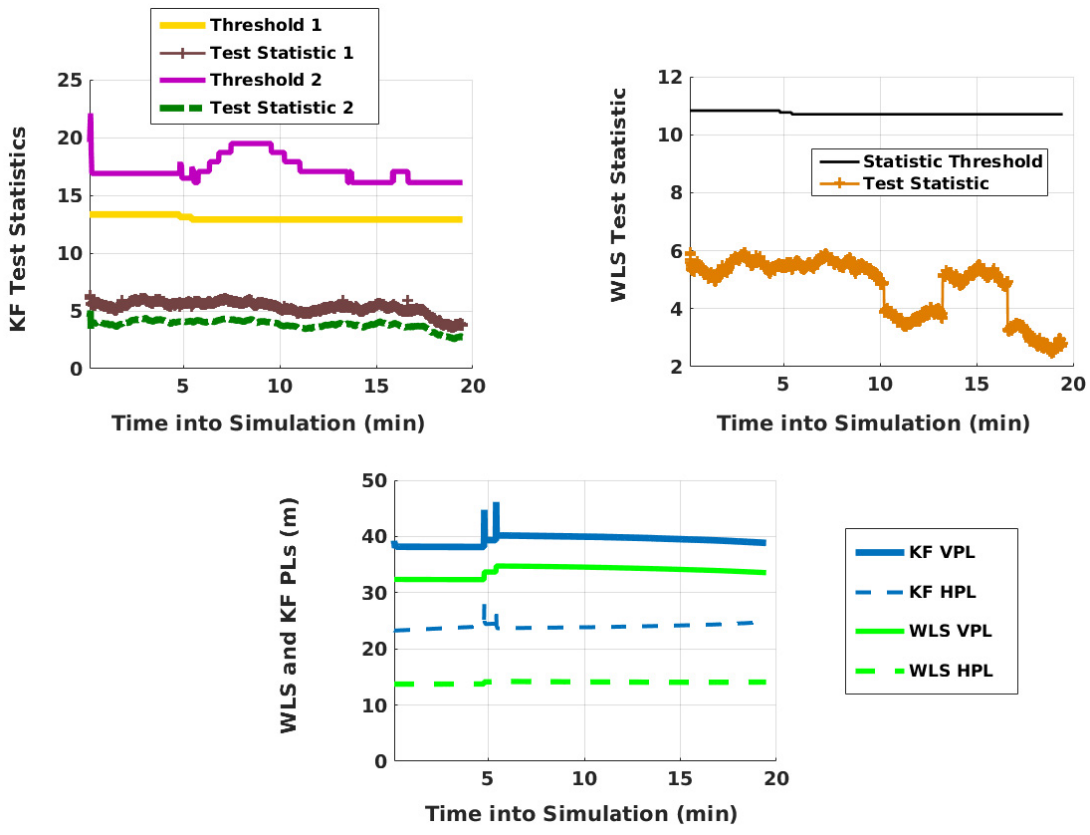
WLS and KF RAIM performance after an initial transient period of 15 s.

Next, the test statistics and PLs of KF RAIM obtained using the SKF are shown alongside those of WLS RAIM in Figure 8. WLS RAIM follows the method of [25] but accounts for the parameter uncertainty of the measurement error model, as discussed in [29]. It is used for comparison purposes for the following reasons: First, it performs fault detection in the range domain, as is with the developed KF RAIM. Second, it is computationally simple. The presented KF RAIM takes 5.5 min in Matlab on an old, low-end IBM Thinkpad laptop for the 19.48 min simulation. The laptop runs on Linux and has 1 GB RAM, 2 GB swap memory, and a Core 2 Duo processor (CPU @ 2 GHz). WLS RAIM, on the other hand, requires 18 s.

**Figure 9:**
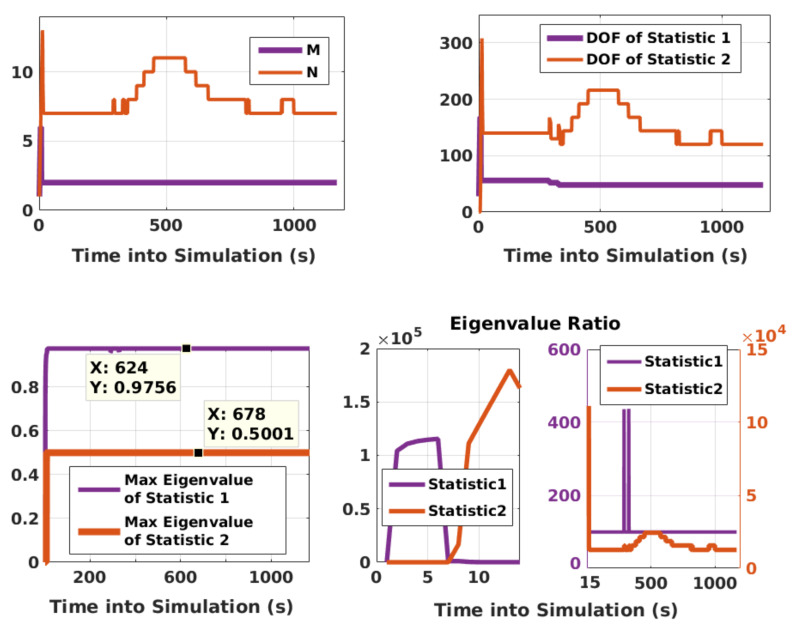
Important parameters of KF RAIM.

In order to gain a good understanding of the properties of KF RAIM, a few salient features are also analyzed. In this regard, Figure 9 depicts the following: *M* (number of terms of the first test statistic $\alpha_{1}$), *N* of Equation (7), maximum eigenvalues, $\lambda_{\Omega ,\;1}^{\max}$ and DOF of the test statistics, and the eigenvalue ratios, $\sqrt{\lambda_{\tilde{\Theta }}^{\max}}/\lambda_{\tilde{\Theta }}^{\min}$. It is evident that $\lambda_{\Omega ,\;1}^{\max}$ of the two statistics is less than one, as desired. Initially, eigenvalue ratios are large because of the addition of white noise during the transient period. The initial transient period is determined by checking the filter innovation sequence. As the eigenvalue ratios have different orders of magnitude, they are subsequently plotted on different scales, each given on either side. The figure also shows adaptive calculations of *M* and *N* and the corresponding changes in the DOF. It is apparent that *M* and *N* must be calculated adaptively as they may change over time.

**Figure 10:**
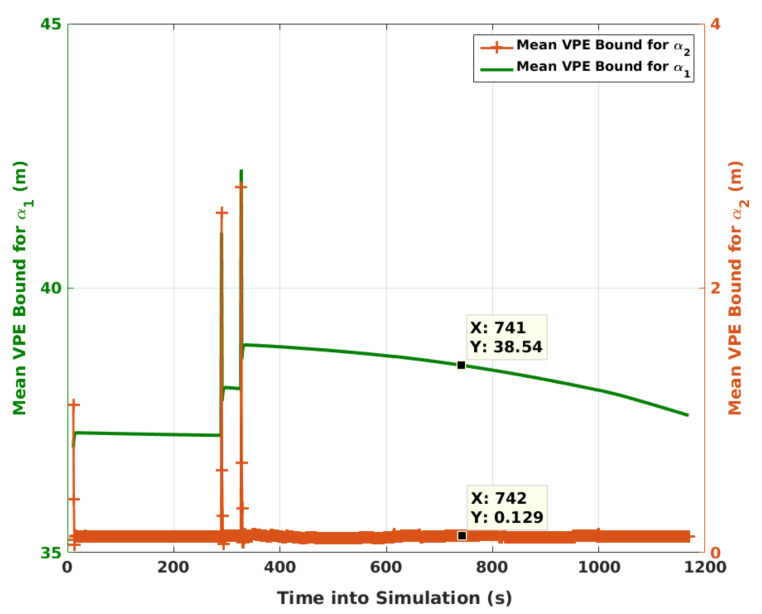
Mean VPE bounds corresponding to $\alpha_{1}$ and $\alpha_{2}$ after an initial transient period of 15 s. It should be noted that the sudden change around 350 s is caused by the change in FMS, not in Γ.

Mean VPE bounds under faults are presented in Figure 10 for $\alpha_{1}$ and $\alpha_{2}$. They are plotted on different y-axes, each on either side. This shows separately the contribution of mean VPE bound corresponding to each test statistic. While *N* is generally much larger than *M*, the second bound with (N−M) terms is much less than the first with *M* terms. Although not shown in the figure, the third bound from ignored terms before *N* recent epochs has an even smaller contribution. It is on the order of 0.02 m. That said, the contributions of all epoch measurements, including those before *N*, to the mean position error bound must be taken into account for PL calculation.

**Figure 11:**
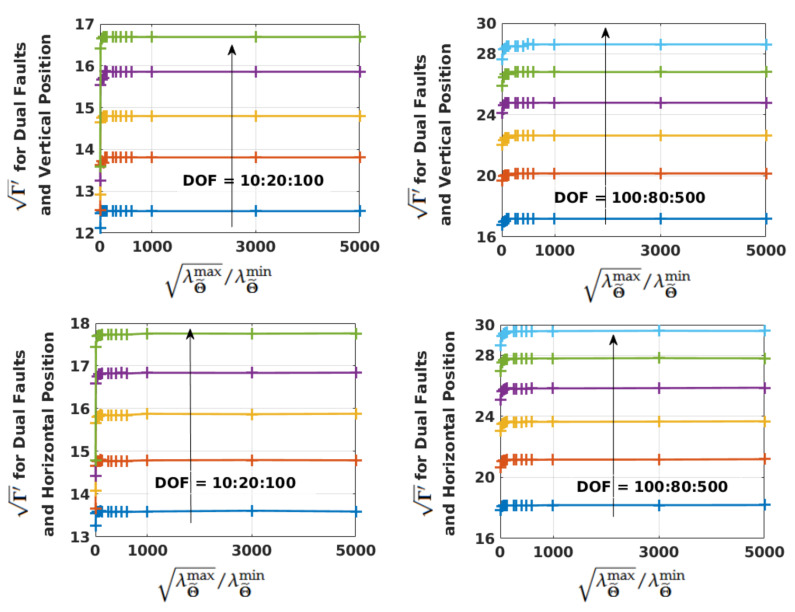
Variation of $\sqrt{\Gamma^{\prime }}$ with $\sqrt{\lambda_{\tilde{\Theta }}^{\max}}/\lambda_{\tilde{\Theta }}^{\min}$ and DOF as a parameter.

Next, $\sqrt{\Gamma^{\prime }}$ defined before Equation (37) is plotted in Figure 11 from the saved lookup table against $\sqrt{\lambda_{\tilde{\Theta }}^{\max}}/\lambda_{\tilde{\Theta }}^{\min}$ with DOF as a parameter. $\sqrt{\Gamma^{\prime }}$ for $P_{MD}^{alloc}$ of both vertical and horizontal positions and dual fault modes is shown. For a given DOF, it levels off at a moderate eigenvalue ratio. Although the figure is plotted up to a ratio of 5000, the trend continues even afterwards. Similar patterns are also seen for the single fault mode. Thus, for a given DOF (or threshold) and $\lambda_{\tilde{\Theta }}^{\max}$, Γ calculated from $\Gamma^{\prime }$ using Equation (39) would not change significantly with large ratios. Therefore, a large $\sqrt{\lambda_{\tilde{\Theta }}^{\max}}/\lambda_{\tilde{\Theta }}^{\min}$ does not imply that the PL would increase to a great extent.

In what follows, fault detection performance with three different navigation filters is discussed.

### 6.2. Fault Detection Performance

Three canonical faults—step, ramp, and sinusoidal—are chosen to analyze fault detection performance. It is explained in [3] that potential failure modes for GNSS precise positioning as complied from the existing literature can be largely modeled as one of the three fault types.

With full-state EKF, the test statistic $\alpha_{EKF,\;k}$ at time step *k* is formed as

(40) $$\begin{array}{cc} \alpha_{EKF,\;k} & =\sum_{\ell =k}^{k-N+1}\left[\Delta {\underline{\rho }}_{\ell }^{T}\;\Delta {\underline{\dot{\rho }}}_{\ell }^{T}\right]R_{\ell }^{-1}\left[\begin{array}{c} \Delta {\underline{\rho }}_{\ell }\\ \Delta {\underline{\dot{\rho }}}_{\ell } \end{array}\right] \end{array}$$

where $R_{\ell }$ is the innovation covariance matrix at time step *ℓ*. $\Delta {\underline{\rho }}_{\ell }$ and $\Delta {\underline{\dot{\rho }}}_{\ell }$ are carrier-smoothed pseudorange and range rate innovation vectors of the full-state EKF. *N* is determined the same way as that in Equation (7), but for the EKF. Under no fault conditions, $\alpha_{EKF,\;k}$ is chi-squared distributed. Its threshold is determined using $P_{FA}$ and DOF. As for WLS and SKF, thresholds are calculated from the non-central chi-squared distribution with the non-centrality parameter 25 for natural biases, following [37]. Test statistics $\alpha_{1}$ and $\alpha_{2}$ each have the probability of false alarm $P_{FA}/2$.

From the PL calculation of WLS RAIM and KF RAIM with SKF, it is noted that simultaneous faults in PRN 28 of GPS (G28 in the skyplot) and PRN 2 of NavIC (N2 in the skyplot) result in maximum mean VPE (or FMS). Hence, faults are injected into these satellites (either N2 or (G28 and N2)) at 100 s into the simulation. In the case of dual fault modes, both satellites have the same fault. Table 1 and Table 2 summarize the fault detection performance for a particular simulation run. Since PLs are not computed for full-state EKF, its change in position error under faulty conditions is only noted. For the –5 m step fault and cosine fault of 5 m amplitude, both $\alpha_{1}$ with SKF and $\alpha_{EKF}$ exhibit an initial peak, but the test statistic of WLS does not. However, the peak of the EKF test statistic crosses its threshold, whereas that with the SKF does not. As a result, no detection of faults occurs with the test statistics of SKF. A possible reason for non-detection is that the magnitude of the faults is close to that of the innovation sequences for SKF, as is evident from Figure 7. It is also important to note that the ramp fault is detected later by both WLS and SKF test statistics when two satellites are faulty as opposed to a single satellite. This is probably because faults in G28 and N2 maximize the FMS for mean VPE, and therefore they take longer times to become detected. The WLS test statistic detects the ramp fault before that of SKF. However, the position error of the latter crosses its PL later.

Next, it is checked if the SKF test statistics can detect step faults in G28 and N2, for which the VPE just crosses the VPL in different simulation runs and at different time instants. It is observed that its first statistic $\alpha_{1}$ exhibits a sharp peak at the onset time of faults. The magnitude of the peak does cross the threshold and is near 45–50, resulting in instant detection. Similar analysis is also performed with WLS. It detects the corresponding step faults within 10 s of PE crossing the PL during initial aircraft descent and before the crossing afterwards. Thus, detection occurs within the required time to alert [41]. The same observation is made with cosine faults.

**Figure 12:**
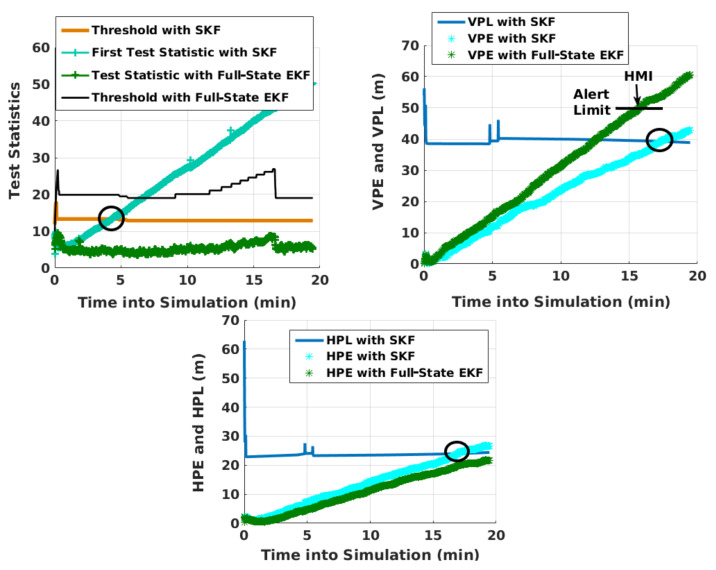
Fault detection with full-state EKF and SKF. Circle indicates when the PE or test statistic of SKF crosses the respective threshold. Vertical alert limit is shown for EKF during the final approach.

Table 1 indicates that the full-state EKF does not detect the ramp faults, but its position error grows with time. With dual faults, the position error increases faster. Although faults were introduced into two satellites of GPS and NavIC—one each—as they maximize the FMS for mean VPE, it is unlikely that satellites from different constellations would fail simultaneously. Therefore, it is investigated what would occur when two satellites of NavIC fail. With NavIC being a new constellation, such a possibility cannot be ruled out. It is noted that if N2 and N6 fail at 10 s into simulation, both with a ramp fault of −0.05 m/s, the EKF VPE crosses the vertical alert limit during the final approach phase without any detection. This is illustrated in Figure 12. The performance of KF RAIM with SKF is also shown for comparison purposes. The EKF test statistic does not display any abnormal behavior because the filter confuses such a slowly growing fault with a residual measurement error that it is supposed to estimate. SKF can detect such faults because it does not estimate measurement errors, but treats them as “consider parameters” instead. Missed detection with full-state EKF is also observed in all of 100 different simulation runs. This occurs with positive slopes as well as different slopes in two satellites.

**Figure 13:**
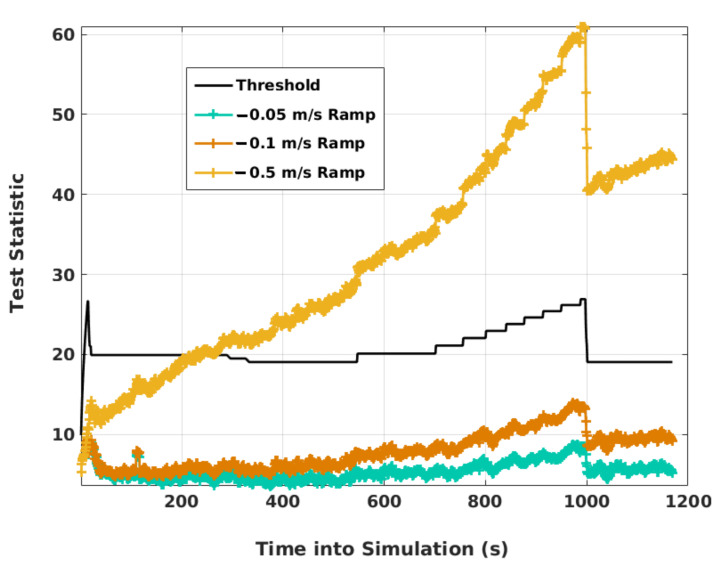
EKF test statistics with different ramp faults.

In order to check if the full-state EKF can detect any other ramp faults, Figure 13 plots $\alpha_{EKF}$ as the magnitude of the fault slope in N2 and N6 is increased. Detection occurs when the slope is −0.5 m/s. Next, to see if detection occurs before the VPE crosses its alert limit or not, faults are injected into N2 and N6 from 895 s in a simulation run. It should be noted that the final approach of the aircraft starts at 911 s and ends around 975 s when the minimum decision altitude of 350 ft is reached. During this period, VPE can be compared against the alert limit of 50 m for EKF. Table 3 shows the performance of EKF and SKF. With −0.5 m/s, $\alpha_{EKF}$ detects faults after the VPE crosses the alert limit, but with the other two slopes, detection occurs before exceeding the alert limit, as desired. Thus, full-state EKF can detect ramp faults successfully when the slope magnitude is greater than a minimum value, which in this case is more than 0.5 m/s. It is also interesting to note that the VPE of EKF grows faster than that of SKF in the presence of ramp faults. As a result, it crosses its alert limit earlier than its counterpart in SKF.

Test results of KF RAIM for a 54.48 min aircraft trajectory are also presented in the Supplementary Materials. The results demonstrate the following: (1) adaptive calculations of all KF RAIM parameters of Figure 9 for long duration data, and (2) the ability of the algorithm to respond to an anomalous behavior and recover from it when the source of the anomaly is gone. The low-end laptop needs 16.3 min to run the KF RAIM algorithm in Matlab. WLS and full-state EKF results are also included. Details of all figures are provided in the description of the Supplementary Materials.

## 7. Conclusions

This paper integrates realistic error models of the carrier-smoothed pseudorange measurements into the RAIM algorithm with an SKF navigation filter. Error models include appropriate time constants of individual error sources, thus overcoming an important limitation of the prior work on KF RAIM. Uncertainties of the model parameters are accounted for. A provision is also made for adding artificial white noise to deal with initial transients in the position error. The suitability of the adopted models for RAIM with SKF is justified through extensive simulations. Performance of the modified KF RAIM is then analyzed with simulated GPS L1 and NavIC L5 signals for aircraft navigation during different phases of flight. The following important conclusions are drawn from the analyses of the results. WLS RAIM has lower PLs than the developed KF RAIM, but it is not appropriate for advanced navigation systems such as vector tracking and sensor integration, where KF is an integral part. A full-state EKF mistakes slow ramp faults for the residual measurement errors it needs to estimate, which results in missed detection. An SKF implementation, on the other hand, can successfully detect such faults. Hence, it offers a promising solution to developing KF RAIM algorithms in the range domain. With realistic error models, the modified KF RAIM is shown to complete processing well within time. Some salient features are also studied to gain insights into its working principles. It is noted that the mean position error bound is mainly determined by a few recent epoch measurements. However, contributions of all epochs must be taken into account for rigorous calculation of the PL for high-reliability applications.

Future work aims at determining error model parameters specific to NavIC measurements, and subsequently validating the KF RAIM with real GPS and NavIC signals and different geometries. Constellation-wide faults need to be handled in the range-based formulation. Furthermore, a fault isolation methodology is required to successfully exclude faulty satellites upon detection. The performance of KF RAIM in weak signal environments will be studied. Its suitability for sensor integration applications will be explored. As ionospheric scintillation prevalent in low latitude regions such as the Indian subcontinent can degrade GNSS measurements and introduce non-Gaussian errors [42,43], it is also important to analyze KF RAIM performance in such scenarios.

## Figures and Tables

**Table 1:** Fault detection performance of SKF and full-state EKF. Fault detection time is calculated from the beginning of simulation. PE stands for position error. It can be horizontal or vertical PE. The corresponding PL is HPL or VPL. Maximum of horizontal PE and VPE is noted under PE. “sat” stands for satellite.

Fault Type	SKF			Full-State EKF		
	Fault Detection Time		If PE > PL	Fault Detection Time		Change in PE
	1 sat (N2)	2 sat (G28 and N2)		1 sat (N2)	2 sat (G28 and N2)	
−5 m step	No	No	No; constant offset	100 s	100 s	Constant offset
	detection	detection	1 sat: PE < 5 m			1 sat: PE < 6.5 m
			2 sat: PE < 10 m			2 sat: PE < 8.55 m
Ramp			Yes	No	No	PE
with slope	563 s	614 s	1 sat: from 1100 s	detection	detection	grows with
−0.05 m/s			2 sat: from 661 s			time
Sinusoidal	No	No	No; sinusoidal PE	No	No	Sinusoidal PE
5sin(2πt/3600) m	detection	detection	1 sat: PE < 5 m	detection	detection	1 sat: PE < 6.5 m
			2 sat: PE < 10 m			2 sat: PE < 10 m
Sinusoidal	No	No	No; sinusoidal PE	100 s	100 s	Sinusoidal PE
5cos(2πt/3600) m	detection	detection	1 sat: PE < 5 m			1 sat: PE < 6.5 m
			2 sat: PE < 10 m			2 sat: PE < 10 m

**Table 2:** Fault detection performance of WLS. Fault detection time is calculated from the beginning of simulation.

Fault Type	WLS		
	Fault Detection Time		If PE > PL
	1 sat (N2)	2 sat (G28 and N2)	
−5 m step	No	No	No; constant offset;
	detection	detection	1 sat: PE < 5 m
			2 sat: PE < 10 m
Ramp			Yes
with slope	495 s	534 s	1 sat: from 943 s
−0.05 m/s			2 sat: from 584 s
Sinusoidal	No	No	No; sinusoidal PE
5sin(2πt/3600) m	detection	detection	1 sat: PE < 5 m
			2 sat: PE < 10 m
Sinusoidal	No	No	No; sinusoidal PE
5cos(2πt/3600) m	detection	detection	1 sat: PE < 5 m
			2 sat: PE < 8 m

**Table 3:** Ramp fault detection performance of full-state EKF and SKF with different slopes. Time is noted from the beginning of simulation.

Fault Slope (m/s)	Full-State EKF		SKF	
	Fault Detection Time (s)	Time When VPE > Alert Limit (s)	Fault Detection Time (s)	Time When VPE > VPL (s)
−0.5	1055	≥975	925	≥999
−0.75	937	≥948	914	≥963
−1	904	≥934.8	909	≥946

**Figure A1:**
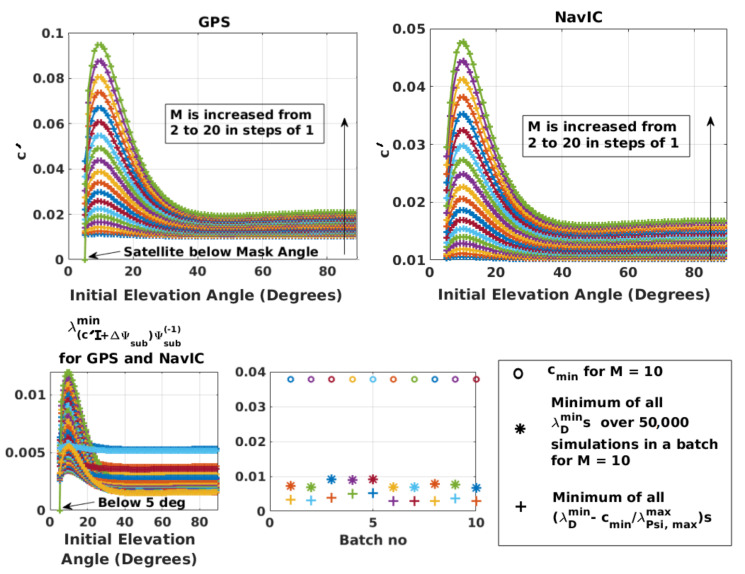
$c^{\prime }$ and $\lambda_{\left(c^{\prime }I+\Delta \Psi_{\mathrm{sub}}\right)\Psi_{\mathrm{sub}}^{-1}}^{\min}$ for different values of *M* and elevation angle (superscript *i* is removed for clarity), and $c_{\min}$ and minimum of $\lambda_{D}^{\min}$s and $\left(\lambda_{D}^{\min}-c_{\min}/\lambda_{\Psi ,\;\max}^{\max}\right)$s for each batch and *M* = 10.

## Data Availability

IF data used for the paper are available from the author upon request. Similar data can also be generated with the details provided in the beginning of Simulation Studies.

## Supplementary Materials

The following are available online at https://www.mdpi.com/article/10.3390/s21248441/s1, matlab_program_figures_1_2.m: Matlab program to reproduce Figure 1 and Figure 2 and simulations described at the end of Section 3; matlab_program_algorithm_1_ts1_figure_3.m: Matlab program for Algorithm A1 in Appendix A for $\alpha_{1}^{\prime }$ and subplots 1 and 2 of Figure 3; matlab_program_algorithm_1_ts2_figure_3.m: Matlab program for Algorithm A1 in Appendix A for $\alpha_{2}^{\prime }$ and subplots 3 and 4 of Figure 3; matlab_program_appendix_figure.m: Matlab program to reproduce the first three subplots of Figure A1; aircraft_trajectory_long_duration.png: 54.48 min aircraft trajectory plot; filter_innovations.png: SKF and full-state EKF innovations and equivalent delta range for WLS; KF_raim_perf1.png: KF test statistics, PLs, and position error plots for long duration data; KF_raim_perf2.png: KF RAIM parameters of Figure 9 for long duration data; wls_full-state_ekf_raim-results.png: WLS and full-state EKF results.

## Appendix A. Algorithm to Justify Use of Ψ Max in Place of Ψ

**Algorithm A1:** Algorithm of simulations to show that $\Psi_{\max}$ can be used in place of Ψ
% Enter constellation specific details and maximum number of visible satellites
% Enter values of *a*${}_{\min}$ and *a*${}_{\max}$ for constellations (e.g., for GPS and NavIC, see
% Figure 2)
for batch no = 1:10 % Ten batches each with 50,000 simulations
for sim no = 1:50,000 % Run 50,000 simulations
% Assume a value for number of epochs (i.e. *M* for $\alpha_{1}^{\prime }$, or (N−M) for $\alpha_{2}^{\prime }$);
% time between two epochs is 1 s; select user speeds and heights within range
% over these epochs
for *i* = 1:total number of satellites
% Pick initially a uniformly random elevation angle $\theta^{\left(1\right)}\left(i\right)$ within 5.1° and
% 90°.
% Pick random scale factors for all τs and error standard deviations
% within range
for *ℓ* = 1: number of epochs – 1
% If *ℓ* == 1, find $\sigma^{\left(\ell \right)}\left(i\right)$; else take the value from previous iteration
% If *ℓ* == 1, assume satellite speed over *M* or (N−M) epochs, as
% described toward the end of Section 3, or keep it constant as it
changes slowly
% Calculate change in elevation angle as $\theta^{\left(\ell +1\right)}\;-\;\theta^{\left(\ell \right)}$ =
% $\pm \;\frac{v_{sat}^{\left(\ell \right)}+v_{user}^{{}^{(}\ell )}}{r_{su}^{\left(\ell \right)}}\times \frac{180}{\pi }$; user height is between 0 and 15 km; select
% ‘+’ or ‘–’ to update θ
% Find $\theta^{\left(\ell +1\right)}\left(i\right)$ by adding/subtracting change in elevation angle
% to/from $\theta^{\left(\ell \right)}\left(i\right)$
% If $\theta^{\left(\ell +1\right)}\left(i\right)$ < 5°, ignore the satellite in subsequent epochs and break
% from *ℓ* loop
% Find $\sigma^{\left(\ell +1\right)}\left(i\right)$
% Calculate $\beta_{\ell q}^{\mathrm{mod}}$ for *q* = 1,…, number of epochs – *ℓ* (see Equation (23))
end % End of *ℓ* loop
% Form $\Psi_{\mathrm{sub}}^{i}$ with $a_{\ell 0}$ and $a_{\ell q}$, and $\Psi_{\mathrm{sub},\;\max}^{i}$ with $a_{\max}$ for satellite *i* over *M*
% or (N−M) epochs
% Check if each element of $\Psi_{\mathrm{sub}}^{i}$≤$a_{\max}^{|\mathrm{row}\;\mathrm{number}\;-\;\mathrm{column}\;\mathrm{number}|\;\mathrm{of}\;\mathrm{the}\;\mathrm{element}}$
% and each element ≥ $K_{\min}{\left(a_{\min}\right)}^{|\mathrm{row}\;\mathrm{number}\;-\;\mathrm{column}\;\mathrm{number}|}$ (Equation (29))
% Check if the minimum eigenvalue of $\Psi_{\mathrm{sub}}^{i}$ is greater than zero
% If any of the previous three conditions is violated, display a message and
% halt
end % End of satellite loop
% Form Ψ and $\Psi_{\max}$; Consider white noise bound in Ψ (Equation (32)), and in
% $\Psi_{\max}$ for $\alpha_{1}^{\prime }$, but not in $\Psi_{\max}$ for $\alpha_{2}^{\prime }$. This is because $\Psi_{\mathrm{sub},\;\max}^{i}$ is
% pre-determined for $\alpha_{2}^{\prime }$ for different (N−M) for its relatively large size, and
% loaded in the RAIM algorithm, while $\Psi_{\mathrm{sub},\;\max}^{i}$ for $\alpha_{1}^{\prime }$ is recursively
% calculated at each time epoch
% Calculate $\Delta \Psi \;=\;(\Psi_{\max}\;-\;\Psi )$ and minimum eigenvalue of D for $\alpha_{1}^{\prime }$,
% where D = $\left(cI\;+\;\Delta \Psi \right)\Psi^{-1}$ (see the last paragraph of Section 4.3 and
% Appendix B)
% Form H matrix. Pick three components of each satellite LOS vector from a
% uniform distribution between –1 and 1 such that the sum of their squares is
% one. Since geometry changes slowly, keep H constant over the number of
% epochs
% Form ${\tilde{S}}^{\prime }$ (see after Equation (4))
% Calculate *c* adaptively for $\alpha_{1}^{\prime }$ so that the maximum eigenvalue of $\Omega_{1}$, $\lambda_{\Omega ,\;1}^{\max}$
% < 1
% Alternatively, calculate *c* as $\left(\lambda_{\Psi ,\;\max}^{\max}-\lambda_{\Psi ,\;\max}^{\min}\right)$ for $\alpha_{2}^{\prime }$ (see after
% Equation (6))
% Calculate Θ as ${\left(cI\;+\;\Psi_{\max}\right)}^{-1}$
% Calculate the minimum and maximum eigenvalues of $\Omega_{1}$ and Ω
% Check if the maximum eigenvalue of Ω, $\lambda_{\Omega }^{\max}$ is ≤ $\lambda_{\Omega ,\;1}^{\max}$
% Check if the minimum eigenvalue of Ω, $\lambda_{\Omega }^{\min}$ is greater than or equal to
% that of $\Omega_{1}\times K_{\min}$ (for $\alpha_{1}^{\prime }$) (and greater than or equal to $\lambda_{\Omega ,\;1}^{\min}\times \frac{K_{\min}}{\left(K_{\mathrm{wh}}\;+\;1\right)}$
% for $\alpha_{2}^{\prime }$)
% Check if $\lambda_{\Omega }^{\min}$ is greater than zero, and if Ω and $\Omega_{1}$ are full rank matrices
% If any of the previous four conditions is violated, display a message and
% halt
end % End of simulation loop
% Plot results of a batch
end % End of batch loop

## Appendix B. Determination of *c*_min_ for $\alpha_{\Omega }^{\prime }$

During the adaptive calculation of *c*, a minimum value, $c_{\min}$ is needed to ensure that $\lambda_{\Omega }^{\max}$ is less than $\lambda_{\Omega ,\;1}^{\max}$ and one, where Ω = ${\tilde{S}}^{\prime T}\Psi {\tilde{S}}^{\prime }{\tilde{S}}^{\prime T}\Theta {\tilde{S}}^{\prime }$ and $\Omega_{1}$ = ${\tilde{S}}^{\prime T}\Psi_{\max}{\tilde{S}}^{\prime }{\tilde{S}}^{\prime T}\Theta {\tilde{S}}^{\prime }$. $c_{\min}$ is considered to be the initial guess of *c*. It is determined in this section. First, the modeled $a_{\ell q}$ of Equation (20) form the elements of Ψ (or sub-matrix $\Psi_{\mathrm{sub}}^{i}$ for satellite *i*). Then, uncertainties in the knowledge of the actual $a_{\ell q}$ are taken into account.

From the definition, the matrix 2-norm of ${\tilde{S}}^{\prime }$ is one and ${\tilde{S}}^{\prime T}{\tilde{S}}^{\prime }$ = I. It is found through simulations that $\lambda_{\Omega }^{\max}$ is close to the maximum eigenvalue of ΨΘ, $\lambda_{\Psi \Theta }^{\max}$ and generally lower than the latter. This can be verified using the Matlab code, matlab_program_algorithm_1_ts−1_figure_3.m, provided in the Supplementary Materials. Thus, $c_{\min}$ will be determined with the help of $\lambda_{\Psi \Theta }^{\max}$ and the maximum eigenvalue of $\Psi_{\max}\Theta$, $\lambda_{\Psi ,\max\Theta }^{\max}$.

Because Ψ, $\Psi_{\max}$, and $\Theta \;(={\left(cI\;+\;\Psi_{\max}\right)}^{-1})$ are block diagonal matrices, $\lambda_{\Psi \Theta }^{\max}$ is $\max_{i}\left\{\mathrm{maximum}\;\mathrm{eigenvalue}\;\mathrm{of}\;\Psi_{\mathrm{sub}}^{i}\Theta_{\mathrm{sub}}^{i}\right\}$ = $\max_{i}\left\{\lambda_{\Psi^{i},\;\mathrm{sub}\Theta^{i},\;\mathrm{sub}}^{\max}\right\}$, where $\Theta_{\mathrm{sub}}^{i}\;=\;{\left(cI+\Psi_{\mathrm{sub},}^{i}\;\max\right)}^{-1}$. This is also true for $\lambda_{\Psi ,\max\Theta }^{\max}$. Hence, $\Psi_{\mathrm{sub}}^{i}$, $\Psi_{\mathrm{sub},\;\max}^{i}$ and $\Theta_{\mathrm{sub}}^{i}$ can be used instead of their full matrices, when appropriate.

It can be easily shown that for any *c* > 0, $\lambda_{\Psi ,\;\max\Theta }^{\max}$ = $\lambda_{\Psi ,\;\max}^{\max}/\left(c\;+\lambda_{\Psi ,\;\max}^{\max}\right)\;$< 1. On the other hand, $\lambda_{\Psi \Theta }^{\max}$ = 1/$\left(\lambda_{D}^{\min}\;+\;1\right)$, where D = $\left(cI\;+\;\Delta \Psi \right)\Psi^{-1}$ and ΔΨ = $\Psi_{\max}$ - Ψ. So, for $\lambda_{\Psi \Theta }^{\max}$< 1, $\lambda_{D}^{\min}$ should be greater than zero. From the structure, it is evident that D is a block diagonal matrix. Its eigenvalues are the same as those of $\Psi^{-1/2}\left(cI\;+\;\Delta \Psi \right)\Psi^{-1/2}$ and hence real. Since with the modeled $a_{\ell q}$ of Equation (20), the trace of ΔΨ (or the sum of eigenvalues) is zero, its minimum eigenvalue is negative. To ensure $\lambda_{D}^{\min}$ > 0, the minimum *c* ($c^{\prime }$) is proposed to be the absolute value of the minimum eigenvalue of ΔΨ or $\left|\min_{i}\lambda_{\Delta \Psi^{i},\;\mathrm{sub}}^{\min}\right|$ plus a non-zero number 0.01. $\Delta \Psi_{\mathrm{sub}}^{i}$ is ($\Psi_{\mathrm{sub},\;\max}^{i}$ – $\Psi_{\mathrm{sub}}^{i}$), and $\lambda_{\Delta \Psi^{i},\mathrm{sub}}^{\min}$ is its minimum eigenvalue.

The first two subplots of Figure A1 illustrate the proposed $c^{\prime }$ for GPS and NavIC satellites as a function of initial elevation angle over *M* epochs and with *M* as a running parameter. As D is a block diagonal matrix, the third subplot of the figure actually shows variations of $\lambda_{D}^{\min}$ with elevation angle and different *M*. It is apparent that with the selected $c^{\prime }$, $\lambda_{D}^{\min}$ is always greater than zero. Although results are shown for decreasing elevation angles (i.e., $\theta^{\left(\ell +1\right)}\left(i\right)\;=\;\theta^{\left(\ell \right)}\left(i\right)\;-\;|\frac{v_{m}^{sat}+v_{m}^{user}}{r_{su}^{\left(\ell \right)}}|\times \frac{180}{\pi }$), similar plots can be obtained when θ increases over *M* epochs. This can be verified with the Matlab program, matlab_program_appendix_figure.m, of the Supplementary Materials. The maximum value of all $c^{\prime }$s for a given *M* considering both GPS and NavIC and all elevation angles is denoted as $c^{\prime \prime }$. In addition, another $c^{\prime }$ denoted as $c^{\prime \prime \prime }$ is calculated as before, but with $\Psi_{\mathrm{sub},\;\min}^{i}$ (or $\Psi_{\min}$) in place of $\Psi_{\mathrm{sub}}^{i}$ (or Ψ). $\Psi_{\mathrm{sub},\;\min}^{i}$ is formed the same way as that of $\Psi_{\mathrm{sub},\;\max}^{i}$, but with $a_{\min}$. The maximum of $c^{\prime \prime }$ and $c^{\prime \prime \prime }$ is $c_{\min}$ for a given *M*. During the adaptive calculation, the initial guess of *c* for $\alpha_{1}^{\prime }$ should be $c_{\min}$ or more. With the selected $c_{\min}$, $\lambda_{D}^{\min}$ is greater than $c_{\min}/\lambda_{\Psi ,\;\max}^{\max}$ for all elevation angles and *M*≤ 20 (see matlab_program_appendix_figure.m), which ensures that $\lambda_{\Psi \Theta }^{\max}$ < $\lambda_{\Psi ,\;\max\Theta }^{\max}$. It can also be shown that for a *c* higher than $c_{\min}$, this inequality holds. This is because the maximum eigenvalue of $\Psi_{\max}$ is greater than that of Ψ (shown in matlab_program_appendix_figure.m for any $\Psi_{\mathrm{sub}}^{i}$) and for any two symmetric matrices $J_{1}$ and $J_{2}$, $\lambda_{J,\;1}^{\min}\;+\;\lambda_{J,\;2}^{\min}\;\leq \;\lambda_{J}^{\min}$, where J = $J_{1}\;+\;J_{2}$ [44]. Setting $J_{1}$ to $\Psi^{-1/2}\left(c-c_{\min}\right)\Psi^{-1/2}$ and $J_{2}$ to $\Psi^{-1/2}\left(c_{\min}I\;+\;\Delta \Psi \right)\Psi^{-1/2}$, the inequality $\lambda_{\Psi \Theta }^{\max}$ < $\lambda_{\Psi ,\;\max\Theta }^{\max}$ can be proved for any other *c* > $c_{\min}$.

Finally, the fourth subplot of Figure A1 is generated with Algorithm 1, accounting for uncertainties in $a_{\ell q}$ for fourteen GPS and seven NavIC satellites. D is calculated with the pre-determined $c_{\min}$ for a given *M*. The asterisk represents the minimum of all $\lambda_{D}^{\min}$s over 50,000 simulations in a batch. The minimum difference between $\lambda_{D}^{\min}$ and $c_{\min}/\lambda_{\Psi ,\;\max}^{\max}$ is also shown for each batch. With the initial *c* set as 0.05 for *M* = 10 (corresponding $c_{\min}$ = 0.03792), Figure 3 depicts that $\lambda_{\Omega }^{\max}$ < $\lambda_{\Omega ,\;1}^{\max}$ and hence is less than one (see the first subplot of the figure). matlab_program_algorithm_1_ts1_figure_3.m can also verify this for changing satellite and user speeds and user heights within limits.
