# Supplementary figures and images for: Performance Analyses of a RAIM Algorithm for Kalman Filter with GPS and NavIC Constellations
Source: Sensors (Basel). 2021 Dec 17;21(24):8441. doi: 10.3390/s21248441 (PMC8708194; doi:10.3390/s21248441)

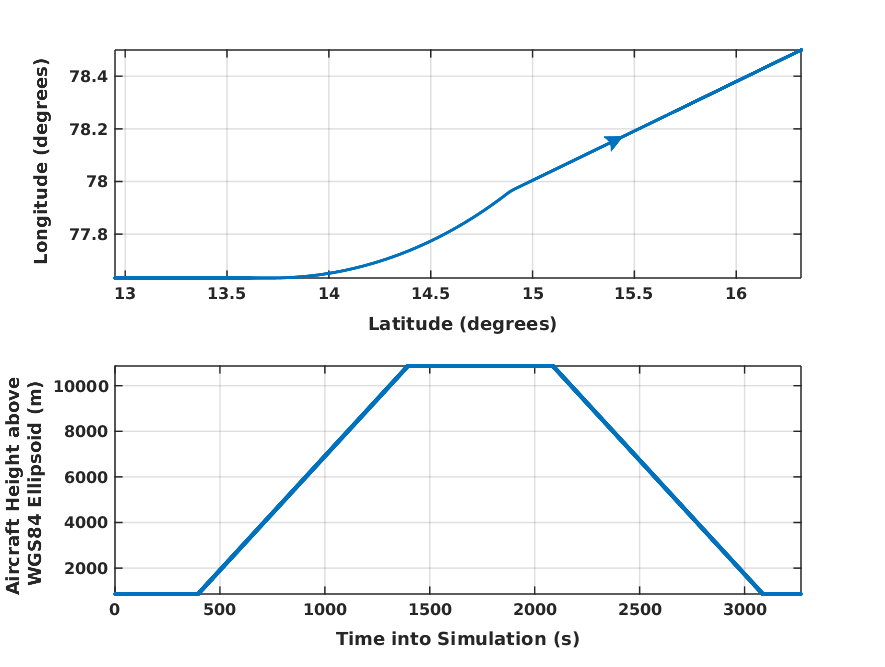

Supplement: Supplementary file 1 [file sensors-21-08441-s001.zip › sensors-1475810-supp-final-done/LongDurationTestResults/aircraft_trajectory_long_duration.png]

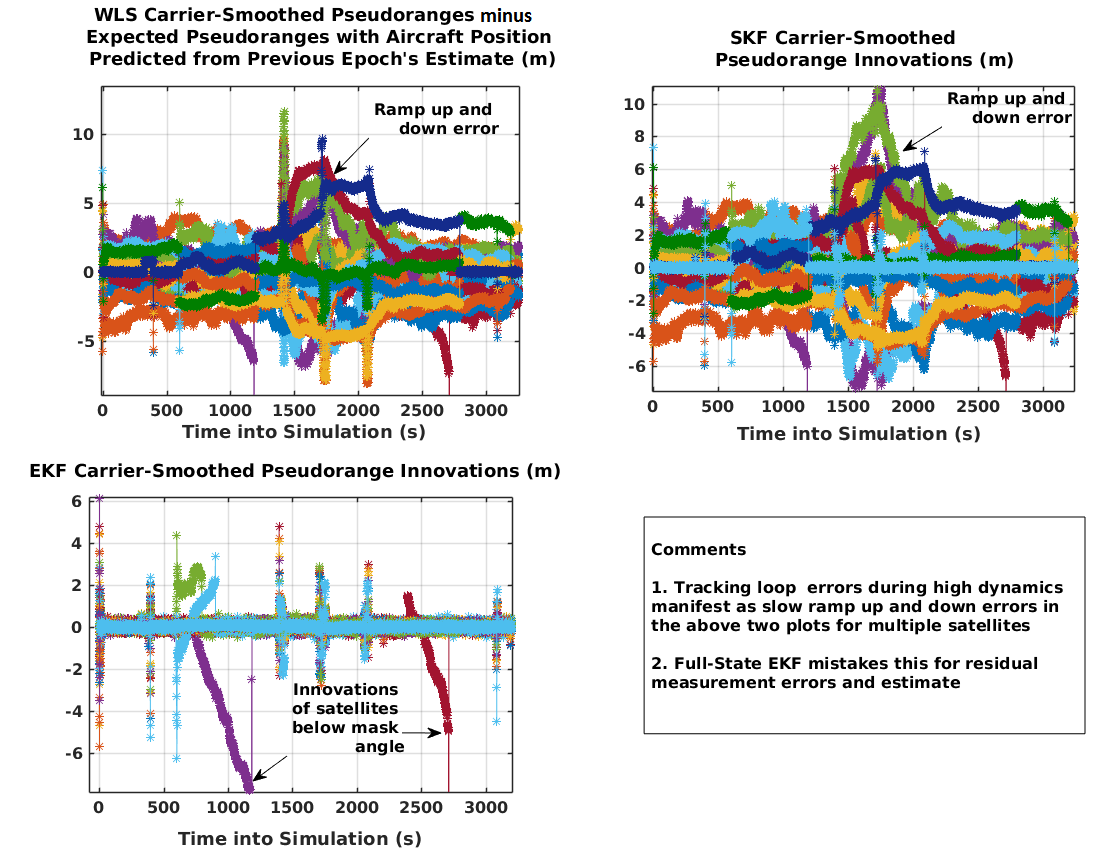

Supplement: Supplementary file 1 [file sensors-21-08441-s001.zip › sensors-1475810-supp-final-done/LongDurationTestResults/filter_innovations.png]

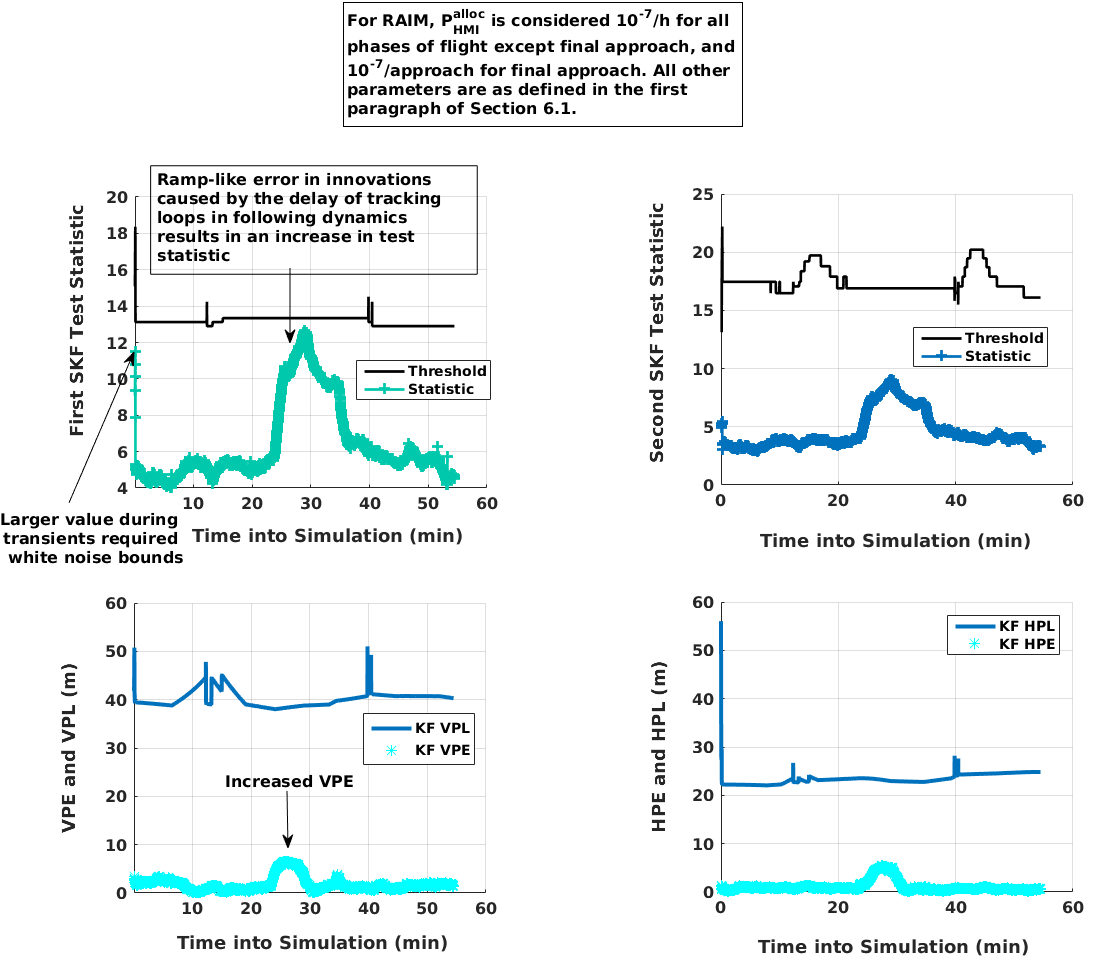

Supplement: Supplementary file 1 [file sensors-21-08441-s001.zip › sensors-1475810-supp-final-done/LongDurationTestResults/kf_raim_perf1.png]

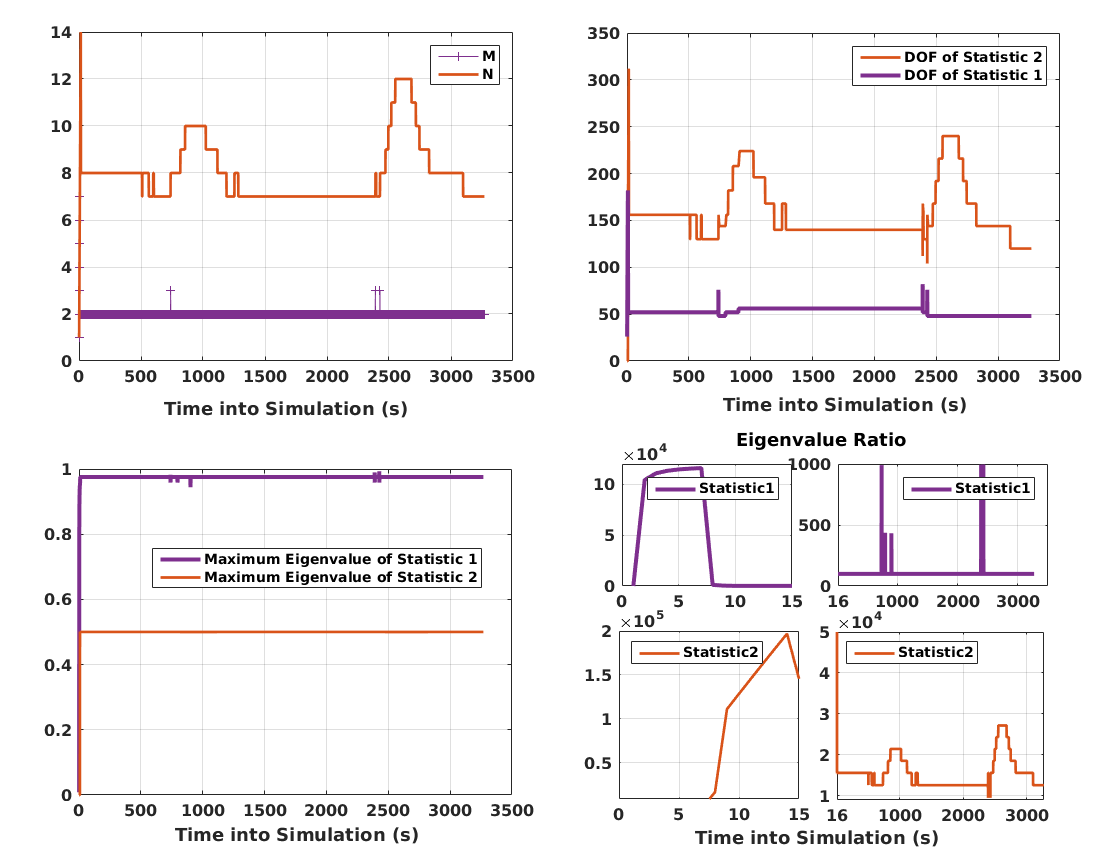

Supplement: Supplementary file 1 [file sensors-21-08441-s001.zip › sensors-1475810-supp-final-done/LongDurationTestResults/kf_raim_perf2.png]

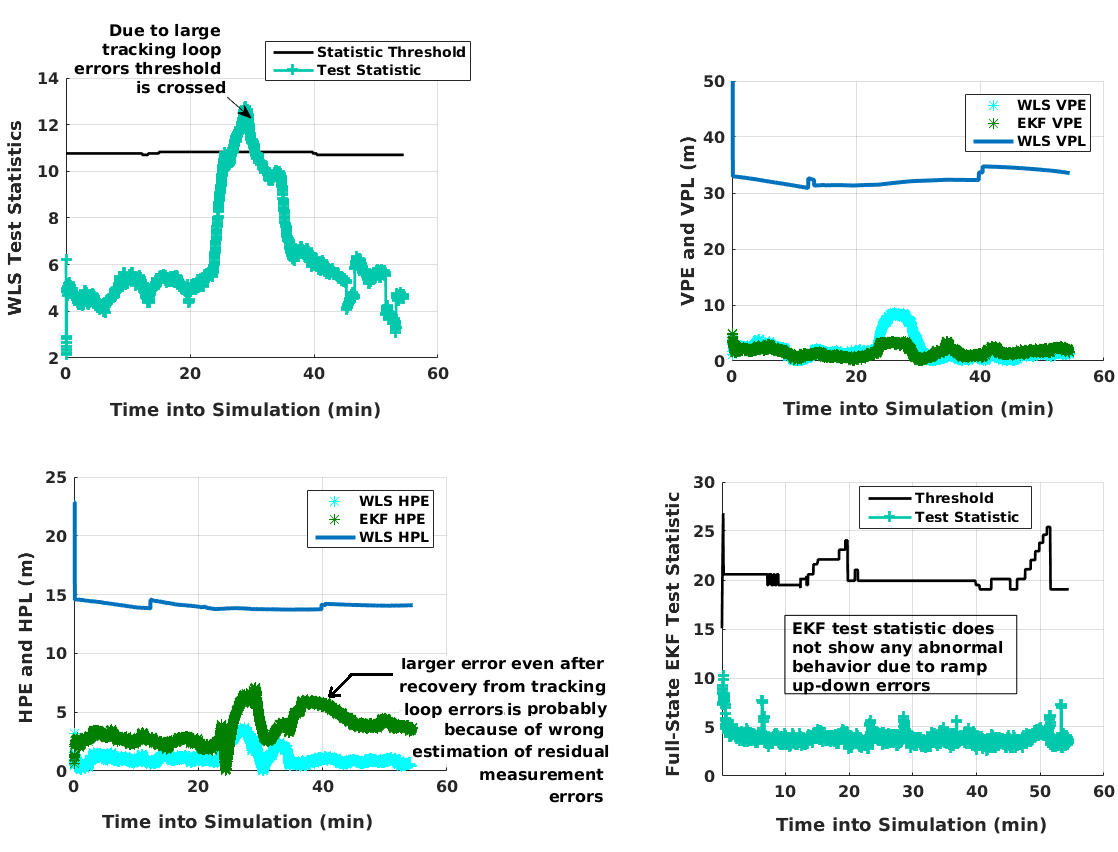

Supplement: Supplementary file 1 [file sensors-21-08441-s001.zip › sensors-1475810-supp-final-done/LongDurationTestResults/wls_full-state_ekf_raim_results.png]
